# Selection of organisms for the co-evolution-based study of protein interactions

**DOI:** 10.1186/1471-2105-12-363

**Published:** 2011-09-12

**Authors:** Dorota Herman, David Ochoa, David Juan, Daniel Lopez, Alfonso Valencia, Florencio Pazos

**Affiliations:** 1 Computational Systems Biology Group, National Centre for Biotechnology (CNB-CSIC), C/Darwin, 3, Cantoblanco, 28049 Madrid, Spain; 2 Structural Bioinformatics Group, Spanish National Cancer Research Centre (CNIO), C/Melchor Fernández Almagro 3, 28029 Madrid, Spain; 3 Centre for Systems Biology (CSB), School of Biosciences, University of Birmingham, Edgbaston, Birmingham, B15 2TT, UK

## Abstract

**Background:**

The prediction and study of protein interactions and functional relationships based on similarity of phylogenetic trees, exemplified by the *mirrortree *and related methodologies, is being widely used. Although dependence between the performance of these methods and the set of organisms used to build the trees was suspected, so far nobody assessed it in an exhaustive way, and, in general, previous works used as many organisms as possible. In this work we asses the effect of using different sets of organism (chosen according with various phylogenetic criteria) on the performance of this methodology in detecting protein interactions of different nature.

**Results:**

We show that the performance of three *mirrortree*-related methodologies depends on the set of organisms used for building the trees, and it is not always directly related to the number of organisms in a simple way. Certain subsets of organisms seem to be more suitable for the predictions of certain types of interactions. This relationship between type of interaction and optimal set of organism for detecting them makes sense in the light of the phylogenetic distribution of the organisms and the nature of the interactions.

**Conclusions:**

In order to obtain an optimal performance when predicting protein interactions, it is recommended to use different sets of organisms depending on the available computational resources and data, as well as the type of interactions of interest.

## Background

There are many computational methods for predicting protein interactions and functional relationships (see [1-3] for recent reviews). Among them, two types of techniques, "phylogenetic profiling" and "similarity of phylogenetic trees", are based on the fact that interacting or functionally related proteins are co-evolving at different levels, defining co-evolution as interdependence between evolutionary histories [4,5].

Phylogenetic profiling [6] is based on the intuitive idea that the genes of two functionally related protein families, which need each other to work, will tend to be both present in the same set of organisms, and probably absent together in the complementary set. A "phylogenetic profile" is a vector representing the pattern of presence/absence of a given gene in a set of organisms, eventually with quantitative information on the sequence similarity of the genes respect to that in a reference organism [7]. Similarity between two of these vectors has been shown to be a good indicator of functional relationship between the families they represent. The similarity of presence/absence patterns between interacting proteins can be seen as a reflection of an extreme case of evolutionary dependence (co-evolution) since the "existence" of the proteins themselves depends on each other.

Co-evolution between interacting or functionally related protein families is also reflected in their phylogenetic trees, being these more similar than expected. Such similarity was first qualitatively evaluated and latter quantified for large collections of interacting and non-interacting protein pairs in order to statistically assess its relationship with interaction [8]. Since then, this idea was applied to study many interacting families, and many groups developed different implementations and variations of the methodology (see [2,4,5] for recent reviews). The basic *mirrortree *methodology for predicting whether two proteins of a given organism interact or not starts by looking for orthologs of these two sequences in a set of genomes. Multiple sequence alignments are then generated for these two sets of orthologs and phylogenetic trees are obtained from them. Pairwise distances are then calculated for all possible pairs of sequences in both sets. Finally, the similarity between these two sets of distances is evaluated with a linear correlation coefficient, using only the distances involving organisms present in both sets. A high correlation coefficient is indicative of similar trees and hence of possible co-evolution. This co-evolutive trend points to a possible interaction or functional relationship between the proteins. This methodology has recently been fully automated and implemented in a web server which allows non-expert users to apply it starting with single sequences [9]. Moreover, this basic methodology has been improved in many ways by different authors (see [4,5] for recent reviews). For example, the background similarity expected between any pair of trees due to the underlying speciation process has been subtracted in different ways in order to improve the predictions [10-12]. More recently, networks representing the pair-wise tree similarities for all proteins in a given genome have been used to improve the prediction of interaction partners and to get insight into the substructure and functioning of macromolecular complexes [13]. Part of this last methodology consist on representing the co-evolutionary context of a given protein by a vector containing its tree similarities (correlation values) with the rest of the proteins, and then re-evaluating the eventual co-evolution between two proteins as the correlation between their corresponding vectors (co-evolutionary profiles). In the same framework of genome-wide co-evolutions, a partial correlation study allows to separate specific from non-specific co-evolutions [13]. It has been shown that these two variants are better predictors of interaction than the original tree correlations.

Both *mirrortree *and "phylogenetic profiling" use a reference set of organisms for looking for orthologs and building the phylogenetic trees or presence/absence profiles respectively. The characteristics of this set (number of organisms, phylogenetic distribution, etc.) are expected to influence the performance of these methodologies. For phylogenetic profiling, some pioneering studies addressed this problem by evaluating the effect of this reference set of genomes on the performance and range of applicability of the methodology [14,15]. Nevertheless, no equivalent study has been done for *mirrortree *and related methodologies. In most studies, the authors use all genomes available in a given resource/database (see references in [4,5]) or, in some cases, they remove redundancy at the strain level [13]. It is worth studying the effect of the organism set in the performance of the *mirrortree*-related methodologies for three main reasons: i) There could be a subset of organisms yielding better results than the whole set of available genomes; ii) different types of interactions (physical, functional, ...) could be better detected using different subsets of organisms; and iii) with the growing number of completely-sequenced genomes, there will be a point in the future were it would not be possible to use all. In such case, it would be valuable to have "recipes" on which subset(s) to use, phrased in terms of number of organisms, phylogenetic distribution, etc.

In this work we explore the effect of using different reference sets of organisms in the performance of the original *mirrortree *algorithm [8] and two of its more recent variants: *profile-correlation *and *context-mirror *[13]. Starting with the set of 214 genomes used by Juan et al. [13], we took different subsets sampled according with different taxonomic criteria, and evaluate the performance of these methodologies using as gold standards sets of interactions of different nature (physical, functional, ...). Our goal is to get insight on the influence of these factors on the co-evolutionary analyses. The results obtained allowed us to propose a number of pragmatic recipes for the use of these methodologies in terms of which subset is better for detecting each particular type of interactions, and which subset to use when the number of available sequenced genomes makes it impossible to use all. Apart from the results obtained from a large scale evaluation, we also show particular examples to illustrate how using different sets of organisms can drastically affect the observed co-evolution between proteins.

## Methods

**Figure 1:**
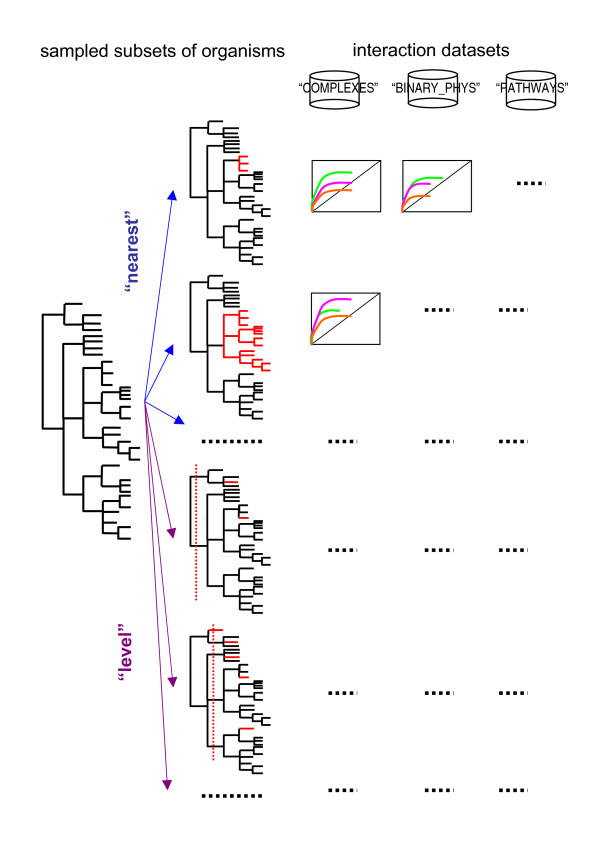
**Schema of the methodology**.From an initial set of organisms with completely sequenced genomes (left), a number of subsets (red) are constructed according with two taxonomic criteria: "nearest" (blue) - following the taxonomy of the reference organism (*E coli K12*) back to the root of the taxonomic tree, all the genomes belonging to each node visited (*E coli *species, Enterobacteriaceae family, etc.) are taken; "level" (purple) - the tree is successively cut at each taxonomic level (superkingdom, phylum, ...) and one organism is taken from each one of the resulting groups (the one with the largest proteome). On the other hand, a number of "gold standard" interaction datasets representing physical and functional interactions of different nature are used (top). For each combination interaction dataset/organism subset, the performance of the three mirrortree-based methodologies is assessed with a partial-ROC analysis (colored curves).

For comparative purposes, we used as initial set of organisms all the Eubacteria and Archaea that were fully sequenced and available in the integr8 database [16] at the time when Juan *et al. *work was performed: 214 genomes. (In that work, redundancy was removed in order not to include very similar organisms, ending up in a final set of 116 organisms.) We then sampled this initial set according with different taxonomic criteria using *E coli K12 *as reference organism, and evaluated the performance of three *mirrortree*-related methodologies in a number of sets representing different types of interactions and using these sampled subsets of organisms as reference sets. Figure 1 illustrates the process.

### Selection of different subsets of organisms

We used the NCBI taxonomic tree [17] as framework for the taxonomy-based selection of organisms. This tree classifies organisms according with a pre-defined hierarchy in which the root represents "cellular organisms", the first level represents the "superkingdoms" (Archaea and Eubacteria in our dataset, which does not include eukarya), the next one the "phylums", and so on. This tree does not contain quantitative information on phylogenetic distances between organisms. Two criteria were used for performing the selections:

• "Nearest". Starting from our reference organism (*E coli K12*) we follow its taxonomy back to the root of the tree and successively take all the organisms belonging to each node. So "nearest_1" represents the *E coli *species (4 organisms -strains-), "nearest_2" contains the Enterobacteriaceae family (21 organisms), and so on up to "nearest_6" which represents the Bacteria superkingdom (195 organisms) and "nearest_7" (whole dataset, bacteria+archaea, 214 organisms). Four organisms are represented in the trees but not used due to the lack of information on their proteomes in the NCBI data. This sampling is designed to evaluate the effect of including close vs. distant organisms in the performance, as well as the effect of the redundancy to some extent (Figure 1).

• "Level". The taxonomic tree is successively cut at each level of the hierarchical classification starting from the root (superkingdom, phylum, ...) and one organism is taken from each resulting group. The criterion for selecting an organism within a group is simply to use that with the highest number of proteins in its genome. The rationale for doing this is to maximize the chances of finding orthologs in that genome in subsequent steps of the process. So, "level_1" would contain 2 organisms (one eubacteria and one archaea), "level_2" contains 16 organisms, one for each phylum. And so on up to "level_9" which represents the whole dataset (214 genomes). This experiment is designed to quantify the effect of sampling homogeneously the taxonomy at different levels of granularity (Figure 1).

• For comparative purposes we also included the set of genomes used in Juan *et al *[13] (116 genomes). This set is very similar to our "level_5" (97 organisms).

Due to the requirement of 15 or more organisms in common between the trees of two protein families (see next point), some of these subsets are never used in practice. The lists of organisms in the final 12 subsets used, as well as representations of their taxonomic distributions, are available in the "Additional file s1".

### Datasets of protein interactions and functional relationships

We used as gold standards to asses the methods' performance three datasets representing *E. coli *protein interactions of different nature and with different characteristics and peculiarities. "PATHWAYS": Functional interactions inferred as co-presence in metabolic pathways taken from the EcoCyc resource [18]. This dataset comprises 4,491 pairs between 719 proteins. "COMPLEXES": Physical interactions (not necessarily direct) inferred by co-presence in macromolecular complexes experimentally determined and taken also from EcoCyc (1,354 pairs between 591 proteins). "BINARY_PHYS": Physical direct binary interactions obtained from the MPIDB database [19]. These have been manually curated from the literature or imported from other databases, providing a high-confidence gold standard to evaluate putative physical direct interactions. The version we used of this database contains 2,103 binary interactions between 1,538 different *E. coli *proteins. The first two datasets were previously used by Juan and co-workers [13], while the last is used here for the first time.

For each dataset, a set of negative examples (proteins assumed not to interact physically or functionally) is constructed by generating all possible pairs between the proteins involved in the positive (interacting) pairs.

### Co-evolution-based prediction of protein interactions

We applied three methods used in Juan *et al. *[13] to predict interacting pairs of proteins using the different sets of reference genomes discussed above for constructing the trees.

The starting point for all methodologies is the generation of phylogenetic trees of orthologs for all *E coli *proteins using the reference sets of organisms sampled as described above. For detecting the ortholog of a given *E coli *protein in each genome we used the "BLAST best bi-directional hit" criterion, with an E-value cutoff of 10E-5, and requiring an alignment coverage of 70%. The orthologs found are aligned with Muscle [20] using the default parameters of this program. Then, a phylogenetic tree is generated from this alignment using the neighbor-joining algorithm implemented in ClustalW [21], excluding the gaps for the distance calculation. A matrix containing the pair-wise distances between all orthologs is generated from this tree by summing the lengths of the branches separating the corresponding leaves.

The *mirrortree *method (MT) evaluates the co-evolution between two proteins by calculating the linear correlation coefficient between their corresponding distance matrices. A minimum of 15 species in common between their trees is required for evaluating a given pair. Only correlation values supported by a (tabulated) P-value of 10E-5 or lower are considered.

A matrix containing the significant pair-wise tree correlations (P-value ≤ 10E-5) for all pairs of proteins within the genome of *E coli *is used as input for the *profile-correlation *method (PC). A row (or column) in this matrix (co-evolutionary profile) contains the correlations between a given protein and all the others in the genome, and can be considered as a representation of the co-evolutionary context for that protein. The *profile-correlation *method re-assesses the co-evolution between two proteins by calculating the linear correlation between their respective co-evolutionary profiles. Finally, the *context-mirror *method (CM) assesses the influence of third proteins in a given co-evolutionary signal observed for two proteins using a partial correlation criterion. This allows separating specific co-evolution (particular to a given pair of proteins) from general co-evolutionary trends involving many proteins. So this method produces results at different "levels" of specificity. See [13] for a more detailed description of these methodologies.

### Evaluation

For each pair of proteins in the *E coli *genome fulfilling the requirements mentioned above, we have the scores of the three methods (*mirrortree*, *profile correlation *and *context-mirror*) based on a given sampled subset of organisms. As commented above, for *context-mirro*r the results are split in different levels of co-evolutionary specificity. In addition, we know whether that pair represents a true interaction or functional relationship according with the datasets described earlier. So, for each combination method/dataset/subset of organisms we have a large list of protein pairs sorted by the score of the method, being each pair labeled as "positive" (the two proteins interact according with the dataset) or "negative" (the two proteins are assumed not to interact).

We apply "receiver operating characteristic" analysis (ROC) [22] to these lists to assess the capacity of the method to separate the positives from the negatives. For each of these lists, the ROC analysis generates a plot of "true positives rate" (TPR) against "false positives rate" (FPR) when varying the classification threshold (score of the method). Curves above the diagonal in this plot represent methods with some discriminative power, being this discriminative capacity better as the curve gets closer to the top-left corner of the plot. Due to the requirement of 15 or more organisms in common in order to evaluate a given pair, the same method applied to the same interaction dataset can produce lists with very different number of pairs (both negatives and positives) when based on different subsets of organisms (trees with different number of leaves). In order to compare the ROC curves in these cases, FPR's and TPR's are calculated respect to the total number of pairs (positives and negatives) in the original dataset, and not respect to the number of pairs rendered by a given subset of organisms. Moreover, defined in this way, these ROC curves give an idea not only on the ability of the method to separate positives and negatives, but also on its range of applicability and coverage: longer curves represent methods that can be applied to (can generate predictions for) a large number of pairs, and the other way around. So, the ROC curves are generated by cutting the sorted list of scores at different thresholds and plotting the resulting TPR's against FPR's calculated as

$$\begin{array}{cc} TPR=Tp∕P=sensitivity\\ FPR=Fp∕N=1-speci\mathrm{fi}city & \end{array}$$

where *Tp*, *Fp *and *Tn *are the true positives, false positives and true negatives obtained at a given threshold, and *P *and *N *the total number of positive and negative pairs for that interaction dataset (irrespective of whether the method could be applied for them with that particular set of organisms or not). Note that these parameters can also be interpreted in terms of "sensitivity" and "specificity" as indicated in the formula above.

Additionally, we also evaluated the results in terms of "precision", "recall" (see Results).

## Results

**Figure 2:**
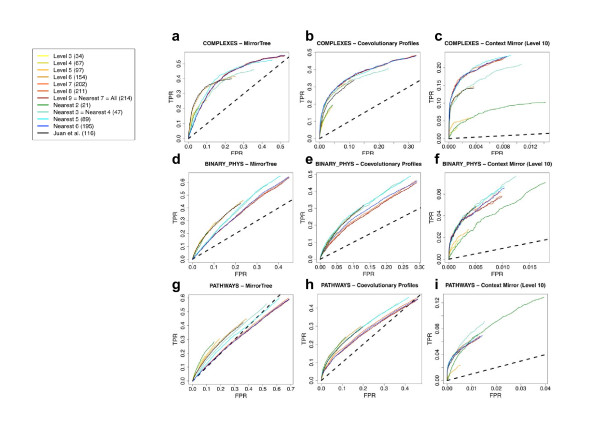
**Matrix of partial ROC curves**. The partial ROC curves evaluate the performance of a given methodology for a given set of interactions using a given set of reference genomes. The rows represent the interaction datasets and the columns the methods. For a given combination method-dataset, the colored curves represent the organism sets according with the included legend. In the legend, the number of organisms within each subset is indicated within brackets. The dotted line highlights the diagonal of the ROC plots, which represents the background performance expected for a random method.

As discussed in detail in Methods, for each combination method/interactions-dataset/subset-of-organisms we obtain a ROC plot which represents the capacity of that method for discriminating interacting from non-interacting pairs of proteins (according with the dataset) when using the phylogenetic trees based on that subset of organisms. Figure 2 shows these ROC plots classified by interaction dataset and method. The different curves within each of these plots correspond to the results obtained with the different organism subsets. For "context-mirror" (CM) we show only the results for level 10, which was shown to represent a good threshold of co-evolutionary specificity for predictive purposes [13]. While the results for other levels vary slightly in terms of accuracy/coverage, their behavior respect to the sets of organisms is virtually identical to those of level 10, and hence they are not included here for the sake of clarity.

Each plot in this figure has its own scale to facilitate the comparison between organism sets, which is the final goal of this work. The same figure with all plots in the same scale, which facilitates the comparison between methods, is available as "Additional file 2". The same results represented in terms of F-measure *vs*. score are available as "Additional file 3", together with an explanation of these parameters. Finally, to have a single numerical estimator of the performance of a given method using the trees derived from a given set of organisms, the maximum F-measure is shown in the "Additional file 4".

In the "Conclusions" section, we derive some recipes for the future use of these methodologies based on the results shown here.

The most obvious observation is that all these co-evolution based methodologies are able to detect a significant number of interactions of different nature across a wide range of organism sets. This is in line with the growing evidence on the relationship between protein interactions and co-evolution, reported by many groups using diverse datasets and variations of the methodology. Another evident observation is that results can vary largely depending on the set of organisms used for building the trees.

PC is the most stable methodology in the sense that its results are those with the lowest dependence on the organism set, as reflected in the highest similarity between ROC curves (except in extreme cases with few organisms) (Figures 2b, e and 2h). It consistently renders good predictions with the highest independence on the organism set. This could be due to the fact that PC is able to filter artefactual tree-correlations such as those related to phylogenetic bias. CM is globally the best methodology and it produces the highest accuracies, but at the expense of requiring a large number of organisms: its results drastically drop off as we use datasets with low number of organisms (Figures 2c, f and 2h). This effect can be easily explained by the fact that CM requires a rich network of significant inter-protein correlations in order to derive partial correlations. Decreasing the number of organisms reduces the chances of obtaining correlations for many pairs (due to the requirement of 15 organisms in common and also the correlation P-value cutoff), which makes such network sparser and less usable for CM. As previously reported, MT is the methodology with the worst performance and, moreover, it is severely affected by the phylogenetic redundancy in the organism set (Figures 2a, d and 2f). In general, the three methods benefit from using datasets with a large number of organisms. However, for MT, this benefit reaches a point where it enters into conflict with the redundancy issue discussed above resulting in "level6" (≈"Juan et al") being the optimal set. PC and CM implicitly correct phylogenetic redundancy and hence they are more benefited when from using more organisms ("nearest6", "nearest7 = level9 = All").

Another global result is that all methods predict better interactions representing co-presence in macromolecular complexes, followed by binary physical interactions, and being co-presence in metabolic pathways the relationships hardest to detect for all (Figure 2). Within this general trend, each type of interaction seems to be better predicted by a certain set of organisms. In general, complexes are better predicted with datasets including phylogenetically distant organisms, while binary interactions and pathways are better predicted with datasets excluding distant organisms: e.g. follow "nearest5", "6" and "7" in Figure 2.

### Examples

We include some examples to illustrate this last result: how using close/distant organisms can drastically affect the predictions. Table 1 contains examples of interactions extracted from the "BINARY_PHYS" dataset which probably correspond to "recent" interactions, as well as others extracted from "COMPLEXES" which probably are "old". The "new" interactions include physical interactions between metabolic enzymes and the interaction between two proteins involved in the division machinery: MinE-MinD [23]. The "old" interactions include some involved in the translation/transcription machinery as well as interactions between ABC transporters. ABC transporters are known to be very ancient systems [24]. The table contains the results that would have been obtained applying the PC method to these cases using as reference sets of organisms "level9" (= all) and "nearest2" (enterobacteriaceae). The correlation coefficient of the PC method indicates the similarity of the co-evolutionary profiles and hence can be seen as a measure of co-evolution. As a measure of performance in detecting the right interactor(s), the "area under the ROC curve" (AUC) [22] is shown. The higher this parameter, the higher are the right interactors (positives) in the sorted list of scores (correlation values). The size of the lists of scores and the number of positives are also indicated. For simplicity and to facilitate the comparison of AUC values, ROC curves are generated here for the positives/negatives which are in the lists, and not taking into account the total number of positives and negatives (as previously done for the ROC curves of Figure 2). It can be seen that "recent" interactions have higher co-evolutionary scores using the "nearest2" dataset than with "level9", and so are the respective predictive performances (AUC). Exactly the opposite happens for the "ancient" interactions: higher co-evolutionary scores and performances are associated to the "level9" set. We follow in detail one of the examples to better understand this table: DPO3A_ECOLI (α subunit of DNA polymerase III) has one reported interaction in the COMPLEXES dataset (with DPO3E_ECOLI, the ε subunit). With the trees constructed based on the "level9" set of organisms, it was possible to apply the PC method to 306 pairs of proteins involving the α subunit (taking into account the requirements and cutoffs described in Methods) one of which is the α-ε pair. Using the "nearest2" set of organisms, it was possible to apply PC to 128 pairs involving DNA pol III α. The co-evolutionary score for α-ε is 0.73 when using the "level9" set of organisms, while it drops to 0.57 when based on "nearest2". As a consequence, there is a much higher proportion of false positives in the sorted list of pairs for "nearest2" compared to "level9" (AUC of 0.11 vs. 0.72). The behavior for the "newer" interactions (e.g. interactions between metabolic enzymes) is exactly the opposite.

**Table 1:** Examples of potentially "new" and "old" interacting pairs of proteins whose co-evolution was evaluated using two sets of organisms

	Protein		Level9(= all)			Nearest2		
			
			Tot/+	AUC	Interactor (corr)	Tot/+	AUC	Interactor (corr)
	MINE_ECOLI	Cell division topological specificity factor	846/1	0.12	MIND_ECOLI (0.52)	223/1	**0.83**	**MIND_ECOLI (0.60)**
	
"recent" (BINARY_PHYS)	PABA_ECOLI	Para-aminobenzoate synthase glutamine amidotransferase component II	671/1	0.28	PABB_ECOLI (0.49)	106/1	**0.96**	**PABB_ECOLI (0.96)**
	
	DHAS_ECOLI	Aspartate-semialdehyde dehydrogenase	760/1	0.17	DNAK_ECOLI (0.48)	384/1	**0.81**	**DNAK_ECOLI (0.90)**
	
	GSHB_ECOLI	Glutathione synthetase	755/1	0.30	AMPM_ECOLI (0.61)	375/1	**0.93**	**AMPM_ECOLI (0.95)**

	DPO3A_ECOLI	DNA polymerase III subunit alpha	306/1	**0.70**	**DPO3E_ECOLI (0.73)**	128/1	0.11	DPO3E_ECOLI (0.57)
	
	DPO3E_ECOLI	DNA polymerase III subunit epsilon	357/1	**0.64**	**DPO3A_ECOLI (0.73)**	123/1	0.22	(0.57) *max*
	
	RPOB_ECOLI	DNA-directed RNA polymerase subunit beta	280/7	**0.82**	**(0.98) *max***	126/4	0.48	(0.93) *max*
	
"old" (COMPLEXES)	RPOA_ECOLI	DNA-directed RNA polymerase subunit alpha	258/6	**0.81**	**(0.80) *max***	90/3	0.48	(0.93) *max*
	
	ZNUB_ECOLI	High-affinity zinc uptake system membrane protein znuB	370/2	**1.00**	**(0.87) *max***	129/1	0.36	ZNUC_ECOLI (0.74)
	
	ZNUC_ECOLI	Zinc import ATP-binding protein ZnuC	386/2	**0.99**	**(0.87) *max***	123/2	0.41	(0.74) *max*
	
	ZNUA_ECOLI	High-affinity zinc uptake system protein znuA	395/2	**0.98**	**(0.87) *max***	39/1	0.79	ZNUC_ECOLI (0.74)

The co-evolution between these proteins was evaluated using the "level9" and "nearest2" sets of organisms. The total number of pairs involving each protein for which it was possible to make calculations, as well as the number of positives (+) are indicated. The co-evolutionary score with the interactor is also shown (corr). For the cases for which the list contain more than one positive the score is the highest one (max). Finally, the AUC value for the list of scores is also included.

## Discussion

Our results show that considerable differences in performance are obtained with mirrortree-based methodologies depending on the set of organisms used for building the trees. They also show that it is not always better to use as many genomes as available, as previously assumed. Most of these results have plausible explanations taking into account the type of interaction and the taxonomic distribution of the organisms.

Although the goal of this work is not to compare methods, but organism sets, our results on the performance of the different *mirrortree *variants are in agreement with previous studies [13]. The lower performance of the baseline MT method compared with PC and CM had been already reported and is related to the fact that these two improved methodologies are able to use the information of genome-wide co-evolutionary networks to better detect real co-evolutions as well as implicitly correct phylogenetic biases [13].

The fact that, in general, all methods work better as more genomes are used is not surprising as more co-evolutive information is available for them. Nevertheless, it is important to take into account the issues related to phylogenetic distances and redundancy commented below. PC and CM to some extent correct tree similarities artificially increased by the introduction of redundant genomes (strains, etc.) [13]. That is not the case for *MT *and hence this methodology is especially sensible to this and other phylogenetic biases, some of which can be corrected explicitly [10,11]. The corrections of all these phylogenetic biases implicit in PC and CM make them to be consistently benefited from using more organisms.

The fact that all methodologies render better results for permanent interactions (macromolecular complexes) had been already reported [13]. Actually, for MT and PC, the results for the binary and pathways datasets, in spite of being clearly significant and different from random, might not be of practical applicability in certain prediction scenarios (i.e. if a high precision is required). The explanation for the better predictions of complexes could be that the evolutionary pressure for co-evolving is expected to be higher in proteins forced to interact permanently than in those with occasional associations. According to these observations macromolecular complexes seem to act as "co-evolutionary units" [13].

Another feature of these macromolecular complexes is that, in general, they represent ancient interactions, compared to transient interactions and functional associations. For this reason, the interaction is expected to occur for all orthologs (interlogs), and hence its associated co-evolutive landmark to be spread through the whole taxonomy. That would explain the observation that better results are obtained for this kind of interactions when including distant organisms within the datasets.

Functional associations and transient interactions are intuitively less prone to yield strong co-evolutions, what would explain the globally lower performances associated to them. Another characteristic of these associations is that, in general, they are "newer" than the macromolecular complexes. It is known that "rewiring" transient interactions is easy and relatively fast in evolutionary terms [25]. For this reason, it may happen that the orthologs of two proteins participating in a transient interaction in a given organism are not interacting in a relatively distant one (they are not true "interlogs") [26,27]. If that is the case, including these "orthologs", which are not interacting and hence not subject to co-evolution, would "dilute" the co-evolutionary signal. This would explain the fact that, for these types of interactions and associations, better results are obtained when using only close organisms, since the interaction is expected to be conserved on them, while it might be absent in taxonomically distant organisms. In other words, many of the *E coli *pathways and transient interactions we are evaluating might be new and hence specific for this microorganism and its close neighbors, and hence the eventual co-evolutions associated to them would be apparent only in these particular genomes. Interestingly, a similar relationship between the "age" of the interactions, their conservation across the taxonomy, and the resulting optimal set of organisms has been reported for the "phylogenetic profiling" method [15].

In some cases it is difficult to disentangle the factors contributing to a given result, for example number of organisms vs. taxonomic criteria used for selecting them. Moreover, it is difficult to quantify and numerically assess the differences of the ROC curves we are using for evaluating performances. For that reason, these curves are evaluated qualitatively and the conclusions presented are based on general trends observed for many curves, instead of particular cases.

A future study aimed at obtaining more insight into the relationship between organism sets and performance should include samplings according with other taxonomic criteria (as well as combinations of them: i.e. combining "nearest"+"level"), and a detailed study of the particular interactions detected and not detected in each experiment (their functional classes, etc).

In the next section, we propose some recipes for the users of these methodologies derived from these results. We plan to implement some of the recipes obtained for the MT method in its recently developed web server [9].

## Conclusion

The number of available genomes continues to grow. And the more we know on protein interactions the more we realize that it is a very complex phenomenon with different types of interactions having different characteristics. For these reasons it is increasingly important to "tune" protein interaction prediction methodologies adapting them for each specific application, instead of using the same protocols and data sources in every situation. Many methods and concepts are being built around the reported relationship between similarity of evolutionary histories (co-evolution) and protein interactions. For this reason it is timely to get insight into the different factors affecting such relationship. Among these factors, a critical one not explored previously is the effect of the organism set used to build the trees on the behavior of these methodologies.

Our results allow us to propose a set of simple and general "recipes" for users on which set of organisms to use depending on the type of interactions they want to predict and the genomic information available.

If phylogenetic trees for the whole genome of interest can be calculated (or are already available in some database/resource), use PC and CM instead of MT. If MT has to be used (i.e. trees not available for all the proteins within a genome, lack of computational resources, etc.) the set of organisms to use should be filtered by phylogenetic redundancy. Filtering at the strain or species level seems to be enough.

PC is a sort of "all-road" method since it shows the lowest dependence on the organism set. It is the best option for general situations, when we are not sure which set of organism to use. It is also better than CM in terms of coverage and hence it is more adequate if we are interested in retrieving many interactions at the expenses of bearing more false positives. Moreover, it is computationally less intensive than CM.

CM should be the chosen option when a lot of genomes (as well as enough computational resources) are available and we are interested in detecting a small number of interactions but highly reliable. Not only it renders the best accuracy but additional information on the structure of the co-evolutionary network which offers some clues about the substructure and functioning of macromolecular complexes is obtained as well [13]. It has to be taken into account that its performance drops drastically when few organisms are available.

Apart from that, if possible it is important to include or exclude distant organisms depending on the type of interactions we try to detect. I.e. to remove phylogenetically distant organisms if we suspect the interactions are not conserved on them ("newer" interactions).

## Competing interests

The authors declare that they have no competing interests.

## Authors' contributions

FP conceived the original idea. FP, DJ and AV designed the experiments. DH, DO and DL implemented the idea and carried out the tests. All authors analyzed the results and contributed writing the manuscript. All authors read and approved the final manuscript.

## Supplementary Material

Additional file 1 **List of organisms in the different subsets and representations of their taxonomic distributions**. Click here for file

Additional file 2 **Version of the Figure 2 with all plots in the same scale**. Click here for file

Additional file 3 Results of Figure 2 given in terms of F-measure (the harmonic mean between "precision" and "recall"). Click here for file

Additional file 4 **Maximum of the F-measure curves of "Additional file **3**"**. This parameter can be regarded as a single numerical estimator of the performance of a given method/organism set, although it does not encompass all the information of a ROC curve. Click here for file
