# Supplementary material for: Selection of organisms for the co-evolution-based study of protein interactions
Source: BMC Bioinformatics. 2011 Sep 12;12:363. doi: 10.1186/1471-2105-12-363 (PMC3179974; doi:10.1186/1471-2105-12-363)
Supplement: Additional file 1 — List of organisms in the different subsets and representations of their taxonomic distributions. [file 1471-2105-12-363-S1.PDF]

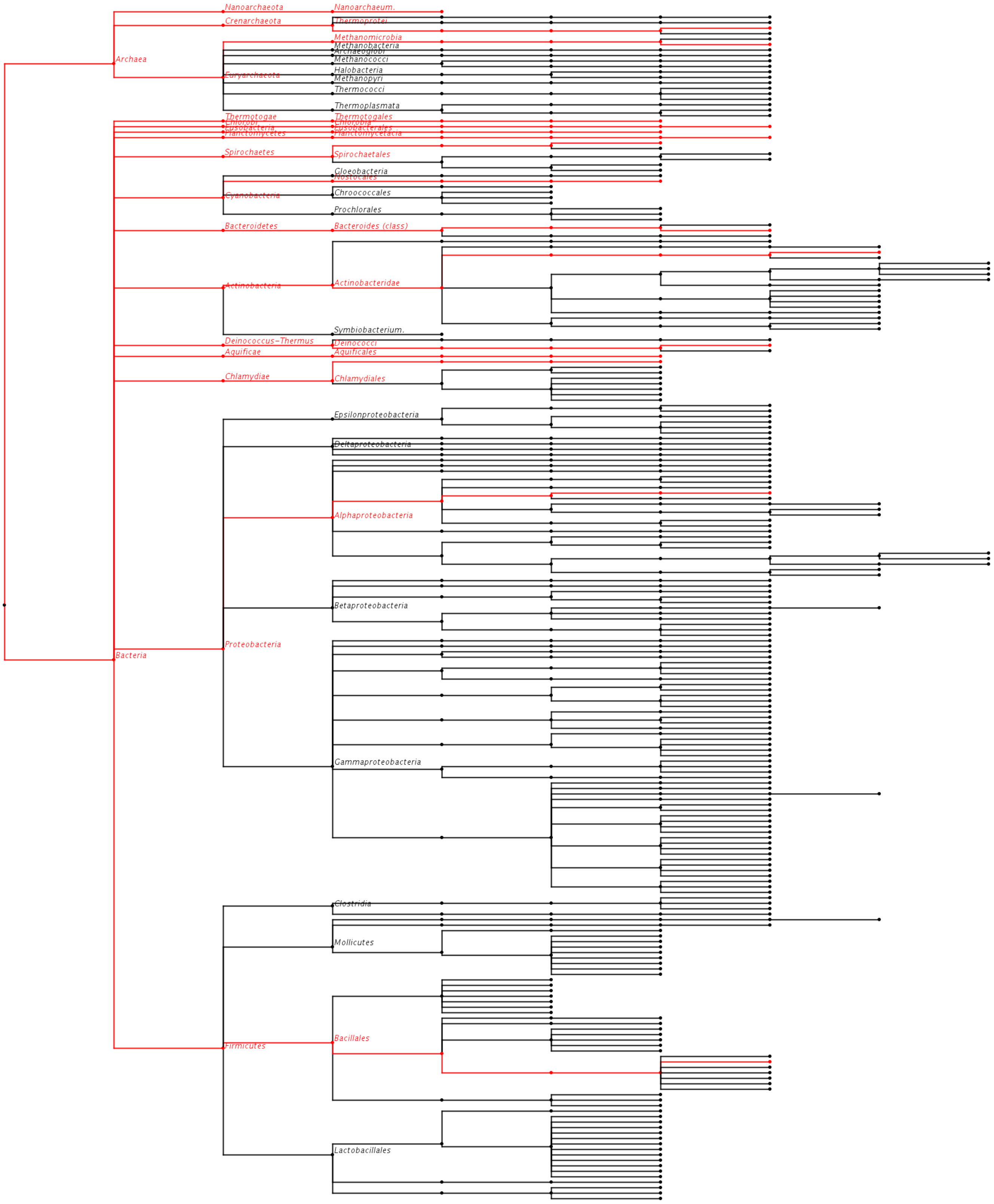
Nanoarchaeota
Nanoarchaeum.
Crenarchaeota
Thermoprotei
Archaea
Methanomicrobia
Methanobacteria
Archaeoglobi
Methanococci
Euryarchaeota
Halobacteria
Methanopyri
Thermococci
Thermoplasmata
Thermotogae
Thermotogales
Chlorobi
Chlorobia
Fusobacteria
Fusobacterales
Planctomycetes
Planctomycetacia
Spirochaetes
Spirochaetales
Gloeobacteria
Nostocales
Cyanobacteria
Chroococcales
Prochlorales
Bacteroidetes
Bacteroides (class)
Actinobacteria
Actinobacteridae
Symbiobacterium.
Deinococcus−Thermus
Deinococci
Aquificae
Aquificales
Chlamydiae
Chlamydiales
Epsilonproteobacteria
Deltaproteobacteria
Alphaproteobacteria
Betaproteobacteria
Bacteria
Proteobacteria
Gammaproteobacteria
Clostridia
Mollicutes
Bacillales
Firmicutes
Lactobacillales

Level 2

Anabaena sp.
Aquifex aeolicus
Bacillus cereus ATCC 10987
Bacteroides thetaiotaomicron
Bradyrhizobium japonicum
Chlorobium tepidum
Fusobacterium nucleatum
Leptospira interrogans lai
Methanosarcina acetivorans
Nanoarchaeum equitans
Parachlamydia sp.
Rhodopirellula baltica
Streptomyces coelicolor
Sulfolobus solfataricus
Thermotoga maritima
Thermus thermophilus HB8

Level 2

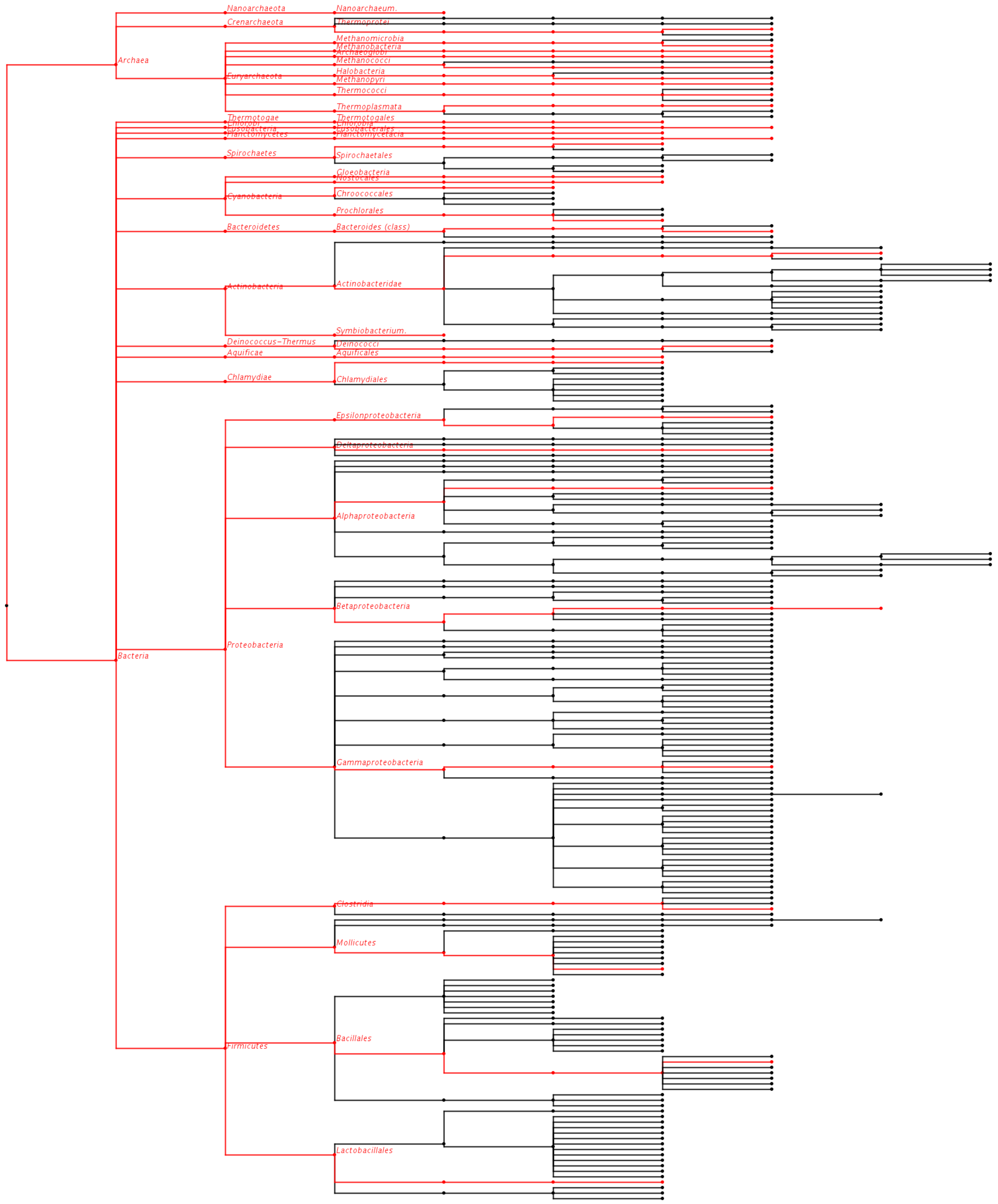
Nanoarchaeota
Nanoarchaeum.
Crenarchaeota
Thermoprotei
Methanomicrobia
Methanobacteria
Archaeoglobi
Methanococci
Archaea
Halobacteria
Euryarchaeota
Methanopyri
Thermococci
Thermoplasmata
Thermotogae
Thermotogales
Chlorobi
Chlorobia
Fusobacteria
Fusobacterales
Planctomycetes
Planctomycetacia
Spirochaetes
Spirochaetales
Gloeobacteria
Nostocales
Cyanobacteria
Chroococcales
Prochlorales
Bacteroidetes
Bacteroides (class)
Actinobacteria
Actinobacteridae
Symbiobacterium.
Deinococcus–Thermus
Deinococci
Aquificae
Aquificales
Chlamydiae
Chlamydiales
Epsilonproteobacteria
Deltaproteobacteria
Alphaproteobacteria
Betaproteobacteria
Bacteria
Proteobacteria
Gammaproteobacteria
Clostridia
Mollicutes
Bacillales
Firmicutes
Lactobacillales

Level 3

Anabaena sp.
Aquifex aeolicus
Archaeoglobus fulgidus
Bacillus cereus ATCC 10987
Bacteroides thetaiotaomicron
Burkholderia pseudomallei
Chlorobium tepidum
Clostridium acetobutylicum
Desulfovibrio vulgaris
Enterococcus faecalis
Fusobacterium nucleatum
Gloeobacter violaceus
Haloarcula marismortui
Leptospira interrogans lai
Methanobacterium thermoautotrophicum
Methanococcus maripaludis
Methanopyrus kandleri
Methanosarcina acetivorans
Mycoplasma penetrans
Nanoarchaeum equitans
Parachlamydia sp.
Picrophilus torridus
Prochlorococcus marinus MIT 9313
Pseudomonas aeruginosa
Pyrococcus horikoshii
Rhizobium loti
Rhodopirellula baltica
Streptomyces coelicolor
Sulfolobus solfataricus
Symbiobacterium thermophilum
Synechocystis sp.
Thermotoga maritima
Thermus thermophilus HB8
Wolinella succinogenes

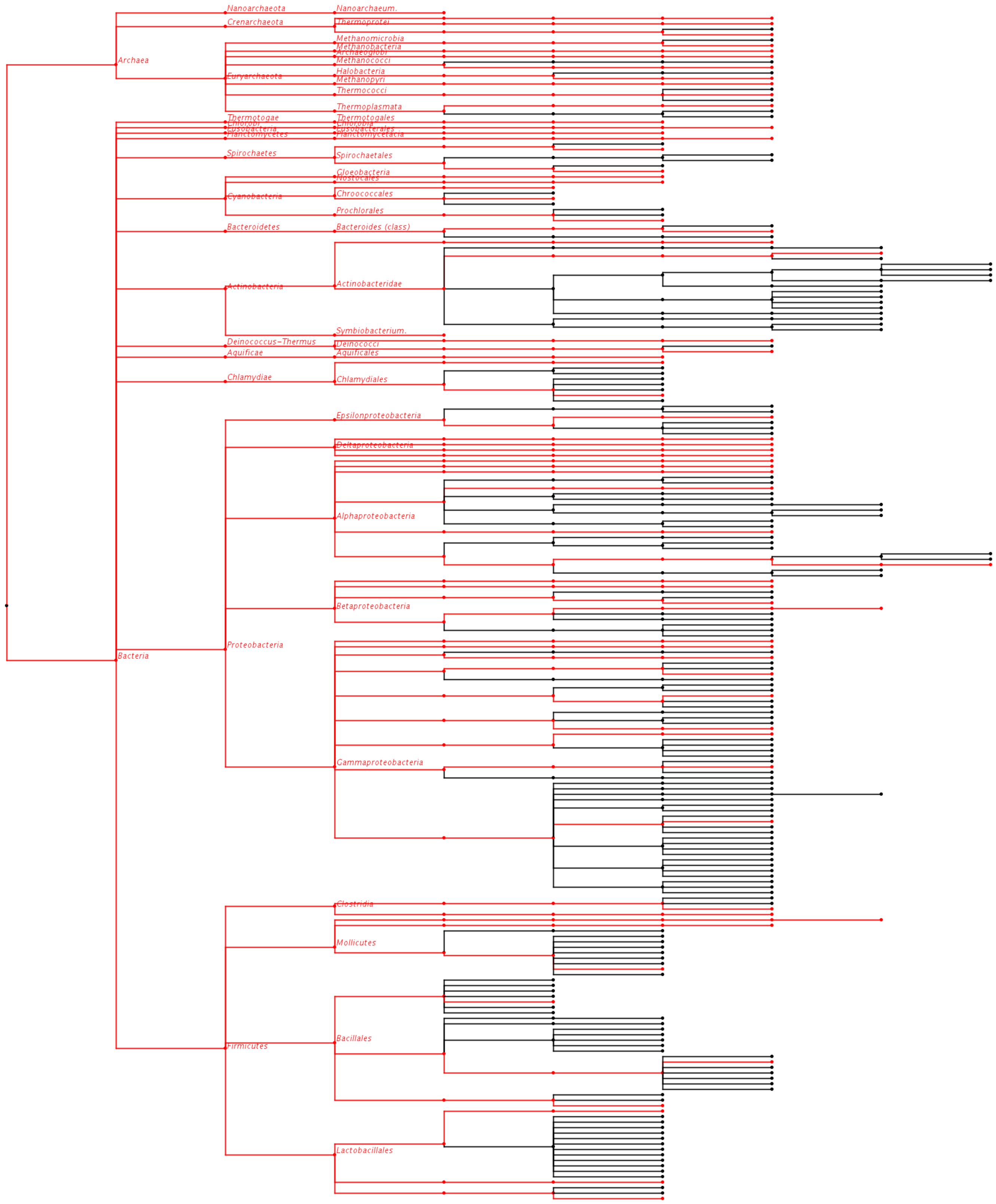
Nanoarchaeota
Nanoarchaeum.
Crenarchaeota
Thermoprotei
Archaea
Methanomicrobia
Methanobacteria
Archaeoglobi
Methanococci
Halobacteria
Euryarchaeota
Methanopyri
Thermococci
Thermoplasmata
Thermotogae
Thermotogales
Chlorobi
Chlorobia
Fusobacteria
Fusobacterales
Planctomycetes
Planctomycetacia
Spirochaetes
Spirochaetales
Gloeobacteria
Nostocales
Cyanobacteria
Chroococcales
Prochlorales
Bacteroidetes
Bacteroides (class)
Actinobacteria
Actinobacteridae
Symbiobacterium.
Deinococcus−Thermus
Deinococci
Aquificae
Aquificales
Chlamydiae
Chlamydiales
Epsilonproteobacteria
Deltaproteobacteria
Alphaproteobacteria
Betaproteobacteria
Bacteria
Proteobacteria
Gammaproteobacteria
Clostridia
Mollicutes
Bacillales
Firmicutes
Lactobacillales

Level 4

Aeropyrum pernix
Anabaena sp.
Aquifex aeolicus
Archaeoglobus fulgidus
Azoarcus sp.
Bacillus cereus ATCC 10987
Bacteroides thetaiotaomicron
Bdellovibrio bacteriovorus
Bifidobacterium longum
Burkholderia pseudomallei
Caulobacter crescentus
Chlamydia pneumoniae TW-183
Chlorobium tepidum
Clostridium acetobutylicum
Deinococcus radiodurans
Desulfotalea psychrophila
Desulfovibrio vulgaris
Enterococcus faecalis
Escherichia coli O6 UPEC
Francisella tularensis
Fusobacterium nucleatum
Geobacter sulfurreducens
Gloeobacter violaceus
Gluconobacter oxydans
Haloarcula marismortui
Lactobacillus acidophilus
Lactococcus lactis
Legionella pneumophila Philadelphia 1
Leptospira interrogans Icterohaemorrhagiae
Listeria innocua
Mesoplasma florum
Methanobacterium thermoautotrophicum
Methanococcus maripaludis
Methanopyrus kandleri
Methanosarcina acetivorans
Methylococcus capsulatus
Mycoplasma penetrans
Nanoarchaeum equitans
Neisseria meningitidis A
Nitrosomonas europaea
Onion yellows phytoplasma
Parachlamydia sp.
Pasteurella multocida
Photobacterium profundum
Picrophilus torridus
Prochlorococcus marinus MIT 9313
Pseudomonas aeruginosa
Pyrobaculum aerophilum
Pyrococcus horikoshii
Rhizobium loti
Rhodopirellula baltica
Rickettsia conorii
Shewanella oneidensis
Silicibacter pomeroyi
Staphylococcus aureus Mu50
Streptomyces coelicolor
Sulfolobus tokodaii
Symbiobacterium thermophilum
Synechococcus sp. PCC 6301
Synechocystis sp.
Thermoanaerobacter tengcongensis
Thermotoga maritima
Thermus thermophilus HB27
Treponema denticola
Wolinella succinogenes
Xanthomonas oryzae
Zymomonas mobilis

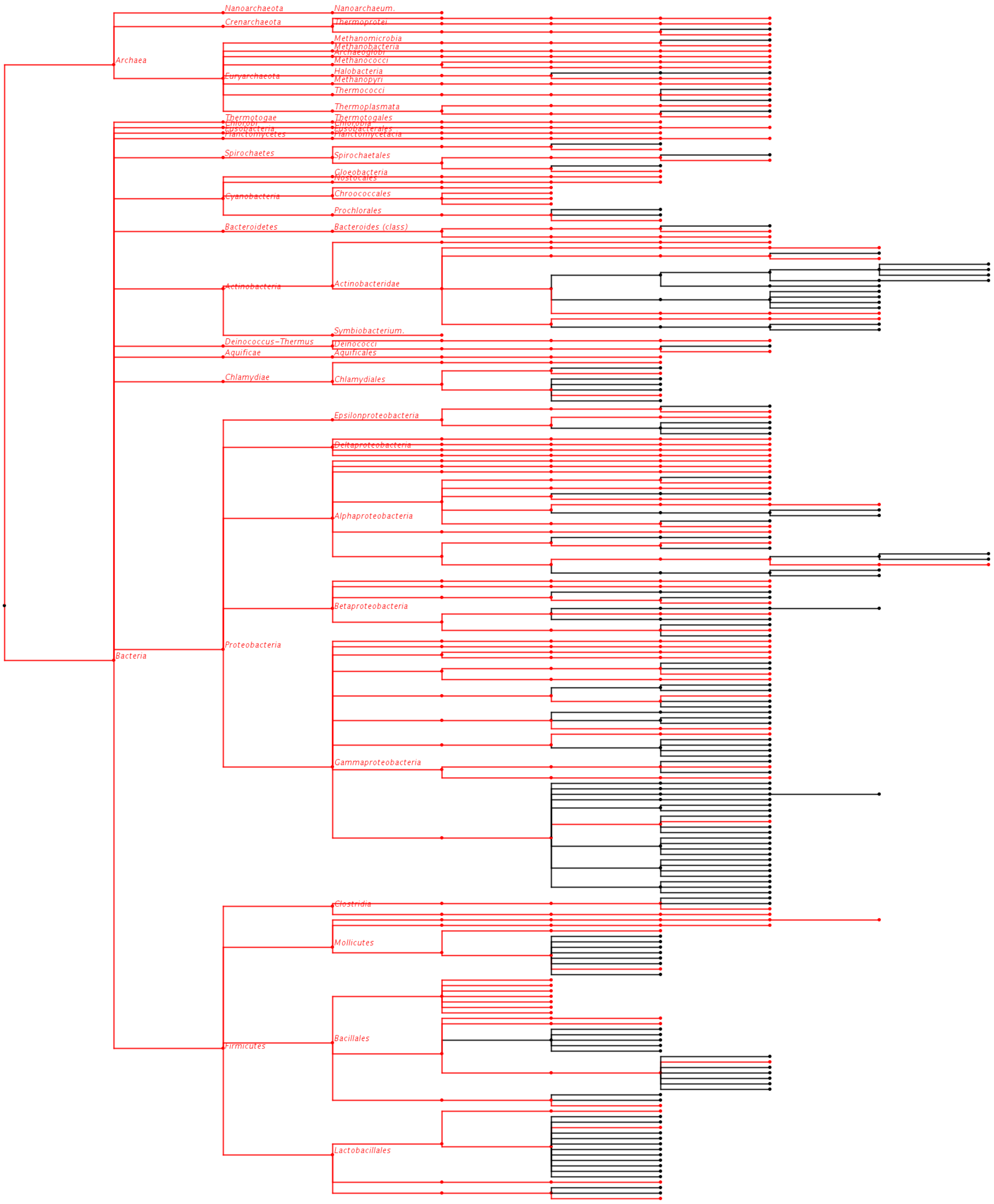
Archaea
Bacteria
Nanoarchaeota
Crenarchaeota
Euryarchaeota
Thermotogae
Chlorobi
Fusobacteria
Planctomycetes
Spirochaetes
Cyanobacteria
Bacteroidetes
Actinobacteria
Deinococcus−Thermus
Aquificae
Chlamydiae
Proteobacteria
Firmicutes
Nanoarchaeum.
Thermoprotei
Methanomicrobia
Methanobacteria
Archaeoglobi
Methanococci
Halobacteria
Methanopyri
Thermococci
Thermoplasmata
Thermotogales
Chlorobia
Fusobacteriales
Planctomycetacia
Spirochaetales
Gloeobacteria
Nostocales
Chroococcales
Prochlorales
Bacteroides (class)
Actinobacteridae
Symbiobacterium.
Deinococci
Aquificales
Chlamydiales
Epsilonproteobacteria
Deltaproteobacteria
Alphaproteobacteria
Betaproteobacteria
Gammaproteobacteria
Clostridia
Mollicutes
Bacillales
Lactobacillales

Level 5

Acinetobacter sp.
Aeropyrum pernix
Anabaena sp.
Aquifex aeolicus
Archaeoglobus fulgidus
Azoarcus sp.
Bacillus cereus ATCC 10987
Bacteroides thetaiotaomicron
Bartonella henselae
Bdellovibrio bacteriovorus
Bifidobacterium longum
Bordetella bronchiseptica
Borrelia garinii
Brucella melitensis
Campylobacter jejuni NCTC 11168
Caulobacter crescentus
Chlamydia muridarum
Chlamydia pneumoniae TW-183
Chlorobium tepidum
Clostridium acetobutylicum
Coxiella burnetii
Deinococcus radiodurans
Desulfotalea psychrophila
Desulfovibrio vulgaris
Ehrlichia ruminantium CIRAD
Enterococcus faecalis
Escherichia coli O6 UPEC
Francisella tularensis
Fusobacterium nucleatum
Geobacillus kaustophilus
Geobacter sulfurreducens
Gloeobacter violaceus
Gluconobacter oxydans
Haloarcula marismortui
Idiomarina loihiensis
Lactobacillus acidophilus
Lactococcus lactis
Legionella pneumophila Philadelphia 1
Leifsonia xyli
Leptospira interrogans Icterohaemorrhagiae
Listeria innocua
Mesoplasma florum
Methanobacterium thermoautotrophicum
Methanococcus jannaschii
Methanococcus maripaludis
Methanopyrus kandleri
Methanosarcina acetivorans
Methylococcus capsulatus
Mycoplasma penetrans
Nanoarchaeum equitans
Neisseria meningitidis A
Nitrosomonas europaea
Nocardia farcinica
Oceanobacillus iheyensis
Onion yellows phytoplasma
Parachlamydia sp.
Pasteurella multocida
Photobacterium profundum
Picrophilus torridus
Porphyromonas gingivalis
Prochlorococcus marinus MIT 9313
Propionibacterium acnes
Pseudomonas aeruginosa
Pyrobaculum aerophilum
Pyrococcus horikoshii
Ralstonia solanacearum
Rhizobium loti
Rhizobium meliloti
Rhodopirellula baltica
Rhodopseudomonas palustris
Rickettsia conorii
Shewanella oneidensis
Silicibacter pomeroyi
Staphylococcus aureus COL
Staphylococcus aureus MRSA252
Staphylococcus aureus MSSA476
Staphylococcus aureus MW2
Staphylococcus aureus Mu50
Staphylococcus aureus N315
Staphylococcus epidermidis ATCC 12228
Streptococcus agalactiae V
Streptomyces avermitilis
Sulfolobus tokodaii
Symbiobacterium thermophilum
Synechococcus elongatus
Synechococcus sp. PCC 6301
Synechococcus sp. WH8102
Synechocystis sp.
Thermoanaerobacter tengcongensis
Thermoplasma volcanium
Thermotoga maritima
Thermus thermophilus HB27
Treponema denticola
Ureaplasma parvum
Wolinella succinogenes
Xanthomonas oryzae
Zymomonas mobilis

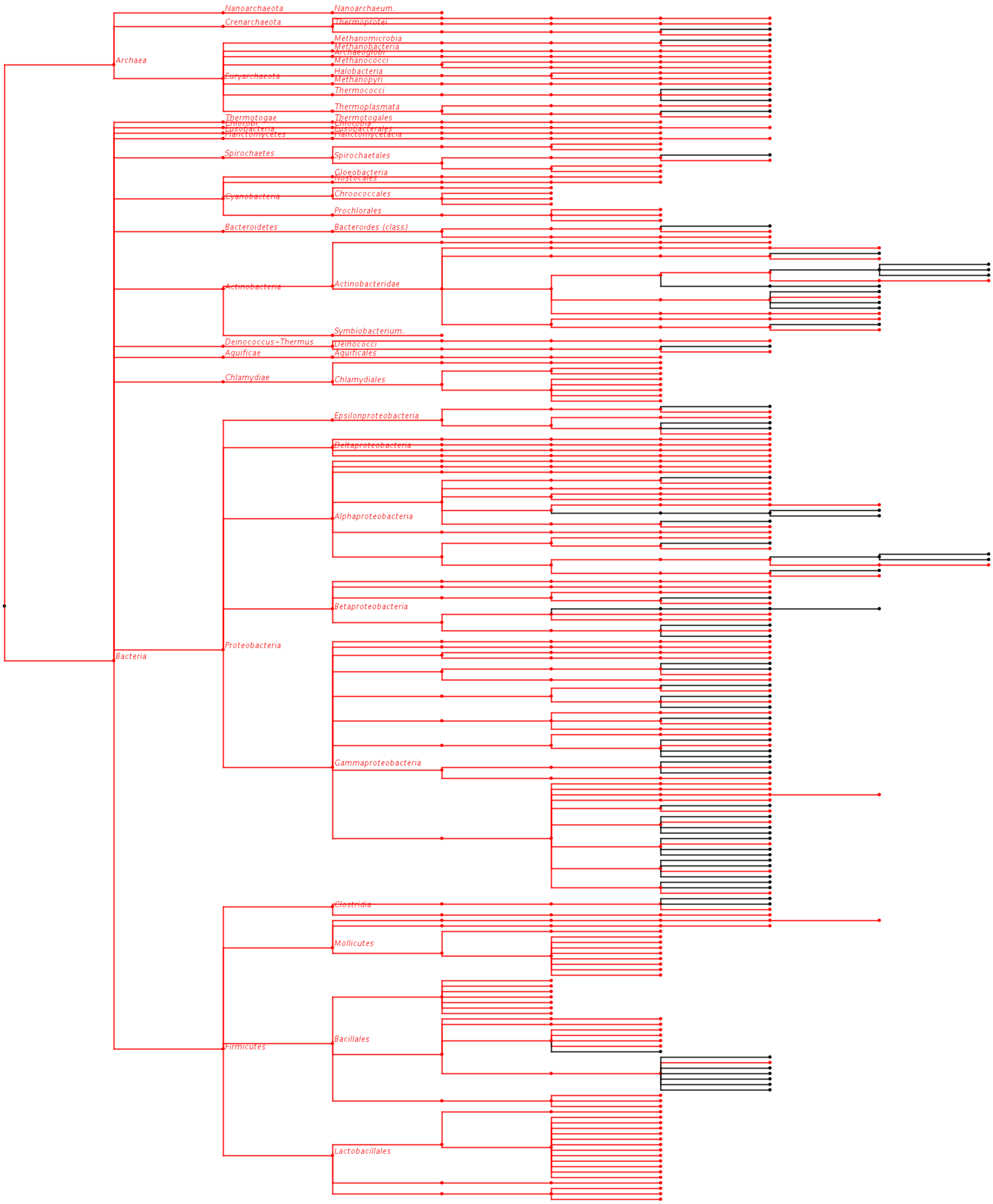

Nanoarchaeota
Nanoarchaeum.
Crenarchaeota
Thermoprotei
Methanomicrobia
Methanobacteria
Archaeoglobi
Methanococci
Archaea
Halobacteria
Euryarchaeota
Methanopyri
Thermococci
Thermoplasmata
Thermotogae
Thermotogales
Chlorobi
Chlorobia
Fusobacteria
Fusobacteriales
Planctomycetes
Planctomycetacia
Spirochaetes
Spirochaetales
Gloeobacteria
Nostocales
Chroococcales
Cyanobacteria
Prochlorales
Bacteroidetes
Bacteroides (class)
Actinobacteria
Actinobacteridae
Symbiobacterium.
Deinococcus−Thermus
Deinococci
Aquificae
Aquificales
Chlamydiae
Chlamydiales
Epsilonproteobacteria
Deltaproteobacteria
Alphaproteobacteria
Betaproteobacteria
Proteobacteria
Bacteria
Gammaproteobacteria
Clostridia
Mollicutes
Bacillales
Firmicutes
Lactobacillales

Level 6

Acinetobacter sp.
Aeropyrum pernix
Anabaena sp.
Anaplasma marginale
Aquifex aeolicus
Archaeoglobus fulgidus
Azoarcus sp.
Bacillus cereus ATCC 10987
Bacillus clausii
Bacillus halodurans
Bacillus licheniformis Goettingen
Bacillus subtilis
Bacteroides thetaiotaomicron
Bartonella henselae
Bdellovibrio bacteriovorus
Bifidobacterium longum
Bordetella bronchiseptica
Borrelia garinii
Bradyrhizobium japonicum
Brucella melitensis
Buchnera aphidicola Acyrthosiphon pisum
Burkholderia mallei
Campylobacter jejuni NCTC 11168
Candidatus Blochmannia floridanus
Caulobacter crescentus
Chlamydia muridarum
Chlamydia pneumoniae AR39
Chlamydia pneumoniae CWL029
Chlamydia pneumoniae J138
Chlamydia pneumoniae TW-183
Chlamydia trachomatis
Chlamydophila caviae
Chlorobium tepidum
Chromobacterium violaceum
Clostridium acetobutylicum
Corynebacterium glutamicum Nakagawa
Coxiella burnetii
Deinococcus radiodurans
Desulfotalea psychrophila
Desulfovibrio vulgaris
Ehrlichia ruminantium Gardel
Enterococcus faecalis
Erwinia carotovora
Escherichia coli O6 UPEC
Francisella tularensis
Fusobacterium nucleatum
Geobacillus kaustophilus
Geobacter sulfurreducens
Gloeobacter violaceus
Gluconobacter oxydans
Haemophilus ducreyi
Haloarcula marismortui
Halobacterium salinarium
Helicobacter pylori ATCC 700392
Idiomarina loihiensis
Lactobacillus acidophilus
Lactobacillus johnsonii
Lactobacillus plantarum
Lactococcus lactis
Legionella pneumophila Philadelphia 1
Leifsonia xyli
Leptospira interrogans Icterohaemorrhagiae
Leptospira interrogans lai
Listeria innocua
Listeria monocytogenes 1/2a
Listeria monocytogenes 4b
Mannheimia succiniciproducens
Mesoplasma florum
Methanobacterium thermoautotrophicum
Methanococcus jannaschii
Methanococcus maripaludis
Methanopyrus kandleri
Methanosarcina acetivorans
Methylococcus capsulatus
Mycobacterium paratuberculosis
Mycoplasma gallisepticum
Mycoplasma genitalium
Mycoplasma hyopneumoniae
Mycoplasma mobile
Mycoplasma mycoides
Mycoplasma penetrans
Mycoplasma pneumoniae
Mycoplasma pulmonis
Nanoarchaeum equitans
Neisseria meningitidis A
Nitrosomonas europaea
Nocardia farcinica
Oceanobacillus iheyensis
Onion yellows phytoplasma
Parachlamydia sp.
Pasteurella multocida
Photobacterium profundum
Photorhabdus luminescens
Picrophilus torridus
Porphyromonas gingivalis
Prochlorococcus marinus CCMP 1375
Prochlorococcus marinus CCMP 1378
Prochlorococcus marinus MIT 9313
Propionibacterium acnes
Pseudomonas aeruginosa
Pyrobaculum aerophilum
Pyrococcus horikoshii
Ralstonia solanacearum
Rhizobium loti
Rhizobium meliloti
Rhodopirellula baltica
Rhodopseudomonas palustris
Rickettsia conorii
Salmonella typhi CT18
Shewanella oneidensis
Shigella flexneri 301
Silicibacter pomeroyi
Staphylococcus aureus COL
Staphylococcus aureus MRSA252
Staphylococcus aureus MSSA476
Staphylococcus aureus MW2
Staphylococcus aureus Mu50
Staphylococcus aureus N315
Staphylococcus epidermidis ATCC 12228
Streptococcus agalactiae III
Streptococcus agalactiae V
Streptococcus mutans
Streptococcus pneumoniae ATCC BAA-255
Streptococcus pneumoniae TIGR4
Streptococcus pyogenes MGAS10394
Streptococcus pyogenes MGAS315
Streptococcus pyogenes MGAS8232
Streptococcus pyogenes SF370
Streptococcus pyogenes SSI-1
Streptococcus thermophilus ATCC BAA-250
Streptococcus thermophilus CNRZ 1066
Streptomyces avermitilis
Sulfolobus tokodaii
Symbiobacterium thermophilum
Synechococcus elongatus
Synechococcus sp. PCC 6301
Synechococcus sp. WH8102
Synechocystis sp.
Thermoanaerobacter tengcongensis
Thermoplasma volcanium
Thermotoga maritima
Thermus thermophilus HB27
Treponema denticola
Treponema pallidum
Tropheryma whipplei Twist
Ureaplasma parvum
Vibrio vulnificus YJ016
Wigglesworthia glossinidia brevipalpis
Wolbachia sp.
Wolinella succinogenes
Xanthomonas axonopodis
Xylella fastidiosa Temecula1
Yersinia pseudotuberculosis
Zymomonas mobilis

Level 6

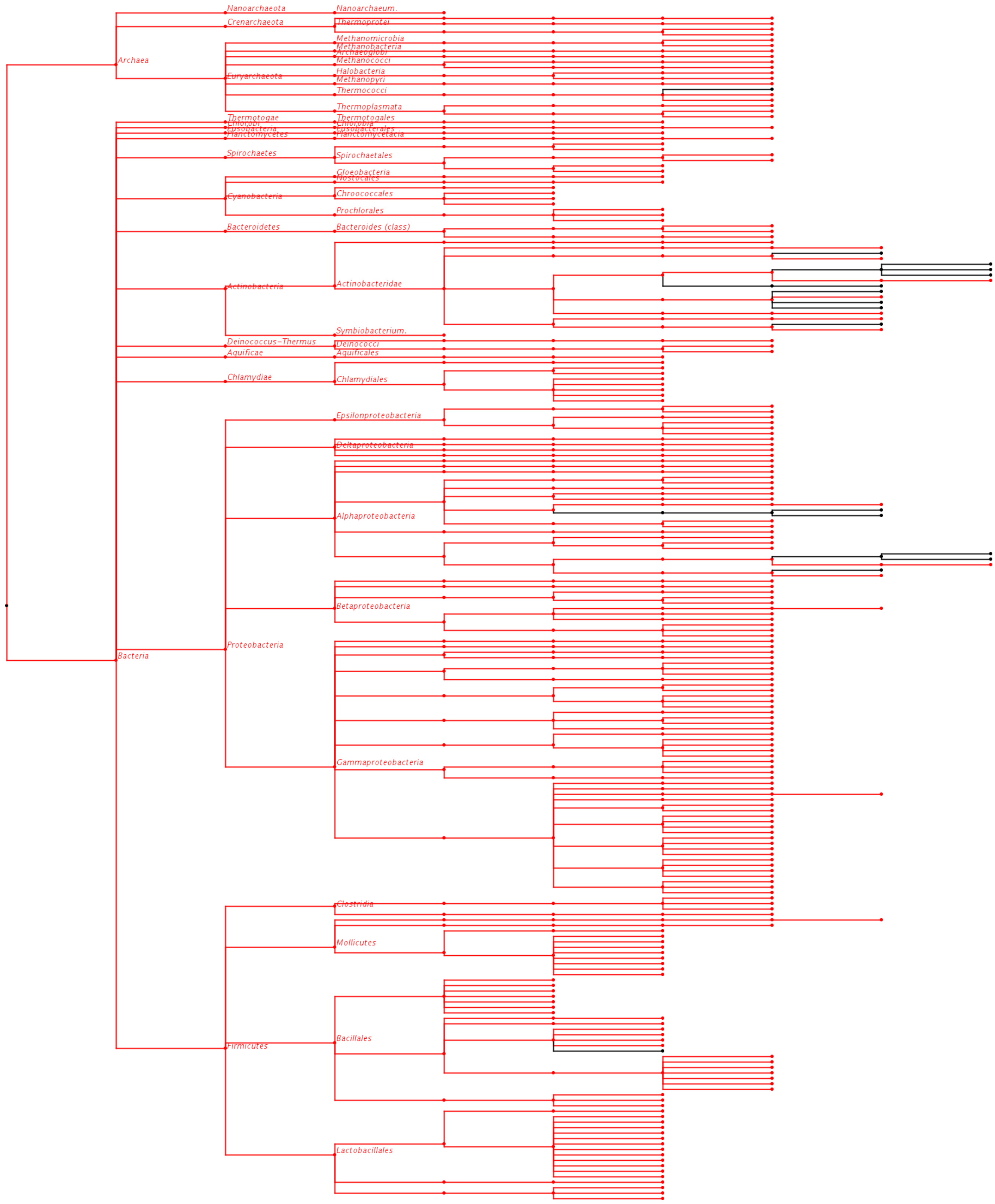
Nanoarchaeota
Nanoarchaeum.
Crenarchaeota
Thermoprotei
Methanomicrobia
Methanobacteria
Archaeoglobi
Methanococci
Halobacteria
Methanopyri
Archaea
Euryarchaeota
Thermococci
Thermoplasmata
Thermotogae
Thermotogales
Chlorobi
Chlorobia
Fusobacteria
Fusobacterales
Planctomycetes
Planctomycetacia
Spirochaetes
Spirochaetales
Gloeobacteria
Nostocales
Cyanobacteria
Chroococcales
Prochlorales
Bacteroidetes
Bacteroides (class)
Actinobacteria
Actinobacteridae
Symbiobacterium.
Deinococcus−Thermus
Deinococci
Aquificae
Aquificales
Chlamydiae
Chlamydiales
Epsilonproteobacteria
Deltaproteobacteria
Alphaproteobacteria
Betaproteobacteria
Bacteria
Proteobacteria
Gammaproteobacteria
Clostridia
Mollicutes
Bacillales
Firmicutes
Lactobacillales

Level 7

Acinetobacter sp.
Aeropyrum pernix
Anabaena sp.
Anaplasma marginale
Aquifex aeolicus
Archaeoglobus fulgidus
Azoarcus sp.
Bacillus anthracis 0581
Bacillus anthracis Porton
Bacillus anthracis Sterne
Bacillus cereus ATCC 10987
Bacillus cereus ATCC 14579
Bacillus cereus ZK
Bacillus clausii
Bacillus halodurans
Bacillus licheniformis Goettingen
Bacillus subtilis
Bacillus thuringiensis
Bacteroides fragilis YCH46
Bacteroides thetaiotaomicron
Bartonella henselae
Bartonella quintana
Bdellovibrio bacteriovorus
Bifidobacterium longum
Bordetella bronchiseptica
Bordetella parapertussis
Bordetella pertussis
Borrelia burgdorferi
Borrelia garinii
Bradyrhizobium japonicum
Brucella melitensis
Brucella suis
Buchnera aphidicola Acyrthosiphon pisum
Buchnera aphidicola Baizongia pistaciae
Buchnera aphidicola Schizaphis graminum
Burkholderia mallei
Burkholderia pseudomallei
Campylobacter jejuni NCTC 11168
Campylobacter jejuni RM1221
Candidatus Blochmannia floridanus
Caulobacter crescentus
Chlamydia muridarum
Chlamydia pneumoniae AR39
Chlamydia pneumoniae CWL029
Chlamydia pneumoniae J138
Chlamydia pneumoniae TW-183
Chlamydia trachomatis
Chlamydophila caviae
Chlorobium tepidum
Chromobacterium violaceum
Clostridium acetobutylicum
Clostridium perfringens
Clostridium tetani
Corynebacterium glutamicum Nakagawa
Coxiella burnetii
Deinococcus radiodurans
Desulfotalea psychrophila
Desulfovibrio vulgaris
Ehrlichia ruminantium CIRAD
Ehrlichia ruminantium Gardel
Enterococcus faecalis
Erwinia carotovora
Escherichia coli EDL933
Escherichia coli K12
Escherichia coli O6 UPEC
Escherichia coli Sakai
Francisella tularensis
Fusobacterium nucleatum
Geobacillus kaustophilus
Geobacter sulfurreducens
Gloeobacter violaceus
Gluconobacter oxydans
Haemophilus ducreyi
Haemophilus influenzae ATCC 51907
Haloarcula marismortui
Halobacterium salinarium
Helicobacter hepaticus
Helicobacter pylori ATCC 700392
Helicobacter pylori J99
Idiomarina loihiensis
Lactobacillus acidophilus
Lactobacillus johnsonii
Lactobacillus plantarum
Lactococcus lactis
Legionella pneumophila Lens
Legionella pneumophila Paris
Legionella pneumophila Philadelphia 1
Leifsonia xyli
Leptospira interrogans Icterohaemorrhagiae
Leptospira interrogans lai
Listeria innocua
Listeria monocytogenes 1/2a
Listeria monocytogenes 4b
Mannheimia succiniciproducens
Mesoplasma florum
Methanobacterium thermoautotrophicum
Methanococcus jannaschii
Methanococcus maripaludis
Methanopyrus kandleri
Methanosarcina acetivorans
Methanosarcina mazei
Methylococcus capsulatus
Mycobacterium paratuberculosis
Mycoplasma gallisepticum
Mycoplasma genitalium
Mycoplasma hyopneumoniae
Mycoplasma mobile
Mycoplasma mycoides
Mycoplasma penetrans
Mycoplasma pneumoniae
Mycoplasma pulmonis
Nanoarchaeum equitans
Neisseria meningitidis A
Neisseria meningitidis B
Nitrosomonas europaea
Nocardia farcinica
Oceanobacillus iheyensis
Onion yellows phytoplasma
Parachlamydia sp.
Pasteurella multocida
Photobacterium profundum
Photorhabdus luminescens
Picrophilus torridus
Porphyromonas gingivalis
Prochlorococcus marinus CCMP 1375
Prochlorococcus marinus CCMP 1378
Prochlorococcus marinus MIT 9313
Propionibacterium acnes
Pseudomonas aeruginosa
Pseudomonas putida
Pseudomonas syringae tomato
Pyrobaculum aerophilum
Pyrococcus furiosus
Pyrococcus horikoshii
Ralstonia solanacearum
Rhizobium loti
Rhizobium meliloti
Rhodopirellula baltica
Rhodopseudomonas palustris
Rickettsia conorii
Salmonella paratyphi-a
Salmonella typhi ATCC 700931
Salmonella typhi CT18
Salmonella typhimurium
Shewanella oneidensis
Shigella flexneri 2457T
Shigella flexneri 301
Silicibacter pomeroyi
Staphylococcus aureus COL
Staphylococcus aureus MRSA252
Staphylococcus aureus MSSA476
Staphylococcus aureus MW2
Staphylococcus aureus Mu50
Staphylococcus aureus N315
Staphylococcus epidermidis ATCC 12228
Streptococcus agalactiae III
Streptococcus agalactiae V
Streptococcus mutans
Streptococcus pneumoniae ATCC BAA-255
Streptococcus pneumoniae TIGR4
Streptococcus pyogenes MGAS10394
Streptococcus pyogenes MGAS315
Streptococcus pyogenes MGAS8232
Streptococcus pyogenes SF370
Streptococcus pyogenes SSI-1
Streptococcus thermophilus ATCC BAA-250
Streptococcus thermophilus CNRZ 1066
Streptomyces avermitilis
Sulfolobus solfataricus
Sulfolobus tokodaii
Symbiobacterium thermophilum
Synechococcus elongatus
Synechococcus sp. PCC 6301
Synechococcus sp. WH8102
Synechocystis sp.
Thermoanaerobacter tengcongensis
Thermoplasma acidophilum
Thermoplasma volcanium
Thermotoga maritima
Thermus thermophilus HB27
Thermus thermophilus HB8
Treponema denticola
Treponema pallidum
Tropheryma whipplei Twist
Ureaplasma parvum
Vibrio cholerae
Vibrio parahaemolyticus
Vibrio vulnificus CMCP6
Vibrio vulnificus YJ016
Wigglesworthia glossinidia brevipalpis
Wolbachia sp.
Wolinella succinogenes
Xanthomonas axonopodis
Xanthomonas campestris campestris
Xanthomonas oryzae
Xylella fastidiosa 9a5c
Xylella fastidiosa Temecula1
Yersinia pestis 91001
Yersinia pestis CO-92
Yersinia pestis KIM5
Yersinia pseudotuberculosis
Zymomonas mobilis

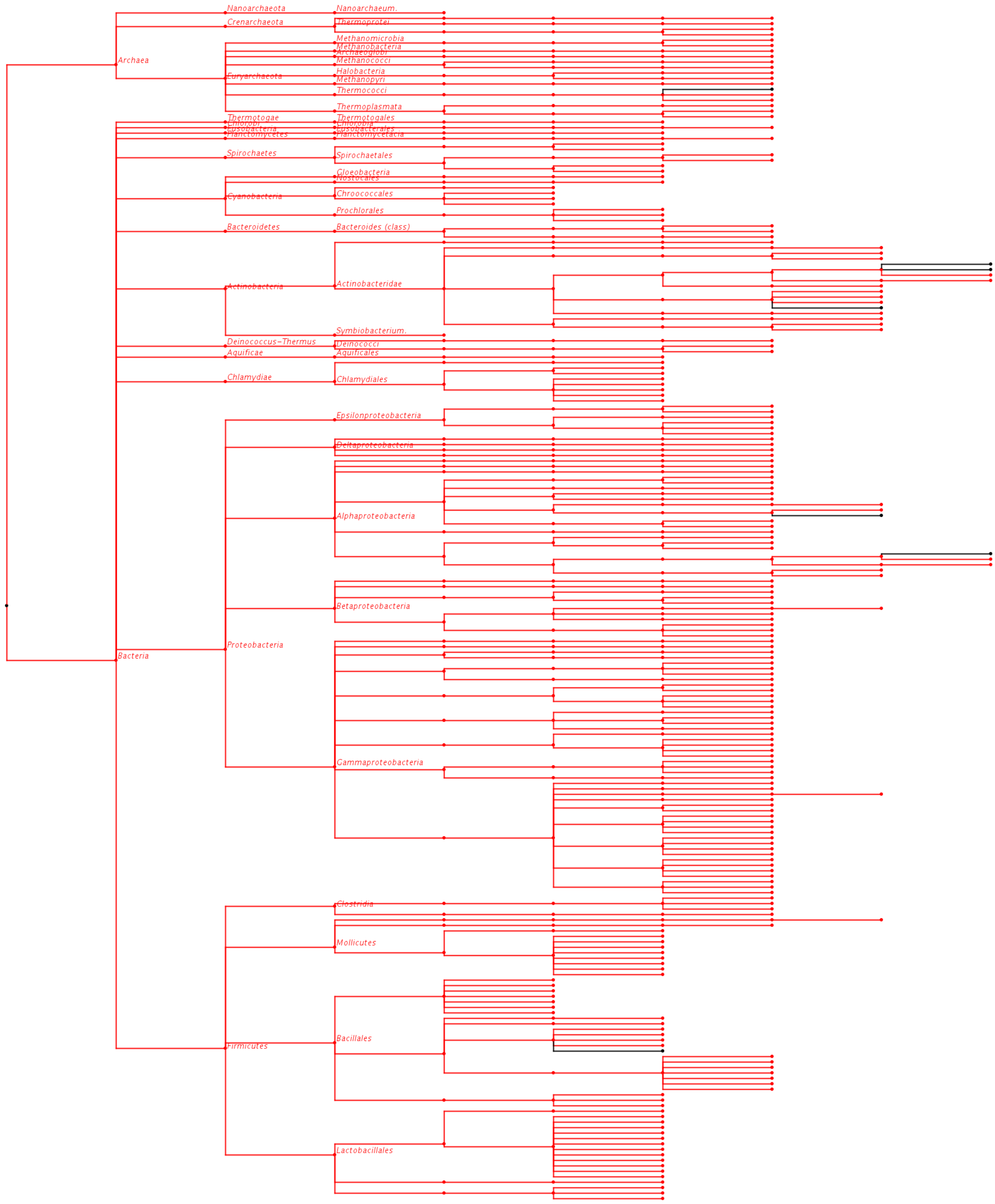
Archaea
Bacteria
Nanoarchaeota
Crenarchaeota
Euryarchaeota
Thermotogae
Chlorobi
Fusobacteria
Planctomycetes
Spirochaetes
Cyanobacteria
Bacteroidetes
Actinobacteria
Deinococcus–Thermus
Aquificae
Chlamydiae
Proteobacteria
Firmicutes
Nanoarchaeum.
Thermoprotei
Methanomicrobia
Methanobacteria
Archaeoglobi
Methanococci
Halobacteria
Methanopyri
Thermococci
Thermoplasmata
Thermotogales
Chlorobia
Fusobacterales
Planctomycetacia
Spirochaetales
Gloeobacteria
Nostocales
Chroococcales
Prochlorales
Bacteroides (class)
Actinobacteridae
Symbiobacterium.
Deinococci
Aquificales
Chlamydiales
Epsilonproteobacteria
Deltaproteobacteria
Alphaproteobacteria
Betaproteobacteria
Gammaproteobacteria
Clostridia
Mollicutes
Bacillales
Lactobacillales

Level 8

Acinetobacter sp.
Aeropyrum pernix
Agrobacterium tumefaciens Dupont
Anabaena sp.
Anaplasma marginale
Aquifex aeolicus
Archaeoglobus fulgidus
Azoarcus sp.
Bacillus anthracis 0581
Bacillus anthracis Porton
Bacillus anthracis Sterne
Bacillus cereus ATCC 10987
Bacillus cereus ATCC 14579
Bacillus cereus ZK
Bacillus clausii
Bacillus halodurans
Bacillus licheniformis Goettingen
Bacillus subtilis
Bacillus thuringiensis
Bacteroides fragilis YCH46
Bacteroides thetaiotaomicron
Bartonella henselae
Bartonella quintana
Bdellovibrio bacteriovorus
Bifidobacterium longum
Bordetella bronchiseptica
Bordetella parapertussis
Bordetella pertussis
Borrelia burgdorferi
Borrelia garinii
Bradyrhizobium japonicum
Brucella melitensis
Brucella suis
Buchnera aphidicola Acyrthosiphon pisum
Buchnera aphidicola Baizongia pistaciae
Buchnera aphidicola Schizaphis graminum
Burkholderia mallei
Burkholderia pseudomallei
Campylobacter jejuni NCTC 11168
Campylobacter jejuni RM1221
Candidatus Blochmannia floridanus
Caulobacter crescentus
Chlamydia muridarum
Chlamydia pneumoniae AR39
Chlamydia pneumoniae CWL029
Chlamydia pneumoniae J138
Chlamydia pneumoniae TW-183
Chlamydia trachomatis
Chlamydophila caviae
Chlorobium tepidum
Chromobacterium violaceum
Clostridium acetobutylicum
Clostridium perfringens
Clostridium tetani
Corynebacterium diphtheriae
Corynebacterium efficiens
Corynebacterium glutamicum Nakagawa
Coxiella burnetii
Deinococcus radiodurans
Desulfotalea psychrophila
Desulfovibrio vulgaris
Ehrlichia ruminantium CIRAD
Ehrlichia ruminantium Gardel
Enterococcus faecalis
Erwinia carotovora
Escherichia coli EDL933
Escherichia coli K12
Escherichia coli O6 UPEC
Escherichia coli Sakai
Francisella tularensis
Fusobacterium nucleatum
Geobacillus kaustophilus
Geobacter sulfurreducens
Gloeobacter violaceus
Gluconobacter oxydans
Haemophilus ducreyi
Haemophilus influenzae ATCC 51907
Haloarcula marismortui
Halobacterium salinarium
Helicobacter hepaticus
Helicobacter pylori ATCC 700392
Helicobacter pylori J99
Idiomarina loihiensis
Lactobacillus acidophilus
Lactobacillus johnsonii
Lactobacillus plantarum
Lactococcus lactis
Legionella pneumophila Lens
Legionella pneumophila Paris
Legionella pneumophila Philadelphia 1
Leifsonia xyli
Leptospira interrogans Icterohaemorrhagiae
Leptospira interrogans lai
Listeria innocua
Listeria monocytogenes 1/2a
Listeria monocytogenes 4b
Mannheimia succiniciproducens
Mesoplasma florum
Methanobacterium thermoautotrophicum
Methanococcus jannaschii
Methanococcus maripaludis
Methanopyrus kandleri
Methanosarcina acetivorans
Methanosarcina mazei
Methylococcus capsulatus
Mycobacterium leprae
Mycobacterium paratuberculosis
Mycobacterium tuberculosis Oshkosh
Mycoplasma gallisepticum
Mycoplasma genitalium
Mycoplasma hyopneumoniae
Mycoplasma mobile
Mycoplasma mycoides
Mycoplasma penetrans
Mycoplasma pneumoniae
Mycoplasma pulmonis
Nanoarchaeum equitans
Neisseria meningitidis A
Neisseria meningitidis B
Nitrosomonas europaea
Nocardia farcinica
Oceanobacillus iheyensis
Onion yellows phytoplasma
Parachlamydia sp.
Pasteurella multocida
Photobacterium profundum
Photorhabdus luminescens
Picrophilus torridus
Porphyromonas gingivalis
Prochlorococcus marinus CCMP 1375
Prochlorococcus marinus CCMP 1378
Prochlorococcus marinus MIT 9313
Propionibacterium acnes
Pseudomonas aeruginosa
Pseudomonas putida
Pseudomonas syringae tomato
Pyrobaculum aerophilum
Pyrococcus furiosus
Pyrococcus horikoshii
Ralstonia solanacearum
Rhizobium loti
Rhizobium meliloti
Rhodopirellula baltica
Rhodopseudomonas palustris
Rickettsia conorii
Rickettsia prowazekii
Salmonella paratyphi-a
Salmonella typhi ATCC 700931
Salmonella typhi CT18
Salmonella typhimurium
Shewanella oneidensis
Shigella flexneri 2457T
Shigella flexneri 301
Silicibacter pomeroyi
Staphylococcus aureus COL
Staphylococcus aureus MRSA252
Staphylococcus aureus MSSA476
Staphylococcus aureus MW2
Staphylococcus aureus Mu50
Staphylococcus aureus N315
Staphylococcus epidermidis ATCC 12228
Streptococcus agalactiae III
Streptococcus agalactiae V
Streptococcus mutans
Streptococcus pneumoniae ATCC BAA-255
Streptococcus pneumoniae TIGR4
Streptococcus pyogenes MGAS10394
Streptococcus pyogenes MGAS315
Streptococcus pyogenes MGAS8232
Streptococcus pyogenes SF370
Streptococcus pyogenes SSI-1
Streptococcus thermophilus ATCC BAA-250
Streptococcus thermophilus CNRZ 1066
Streptomyces avermitilis
Streptomyces coelicolor
Sulfolobus solfataricus
Sulfolobus tokodaii
Symbiobacterium thermophilum
Synechococcus elongatus
Synechococcus sp. PCC 6301
Synechococcus sp. WH8102
Synechocystis sp.
Thermoanaerobacter tengcongensis
Thermoplasma acidophilum
Thermoplasma volcanium
Thermotoga maritima
Thermus thermophilus HB27
Thermus thermophilus HB8
Treponema denticola
Treponema pallidum
Tropheryma whipplei TW08/27
Tropheryma whipplei Twist
Ureaplasma parvum
Vibrio cholerae
Vibrio parahaemolyticus
Vibrio vulnificus CMCP6
Vibrio vulnificus YJ016
Wigglesworthia glossinidia brevipalpis
Wolbachia pipientis wMel
Wolbachia sp.
Wolinella succinogenes
Xanthomonas axonopodis
Xanthomonas campestris campestris
Xanthomonas oryzae
Xylella fastidiosa 9a5c
Xylella fastidiosa Temecula1
Yersinia pestis 91001
Yersinia pestis CO-92
Yersinia pestis KIM5
Yersinia pseudotuberculosis
Zymomonas mobilis

Level 8

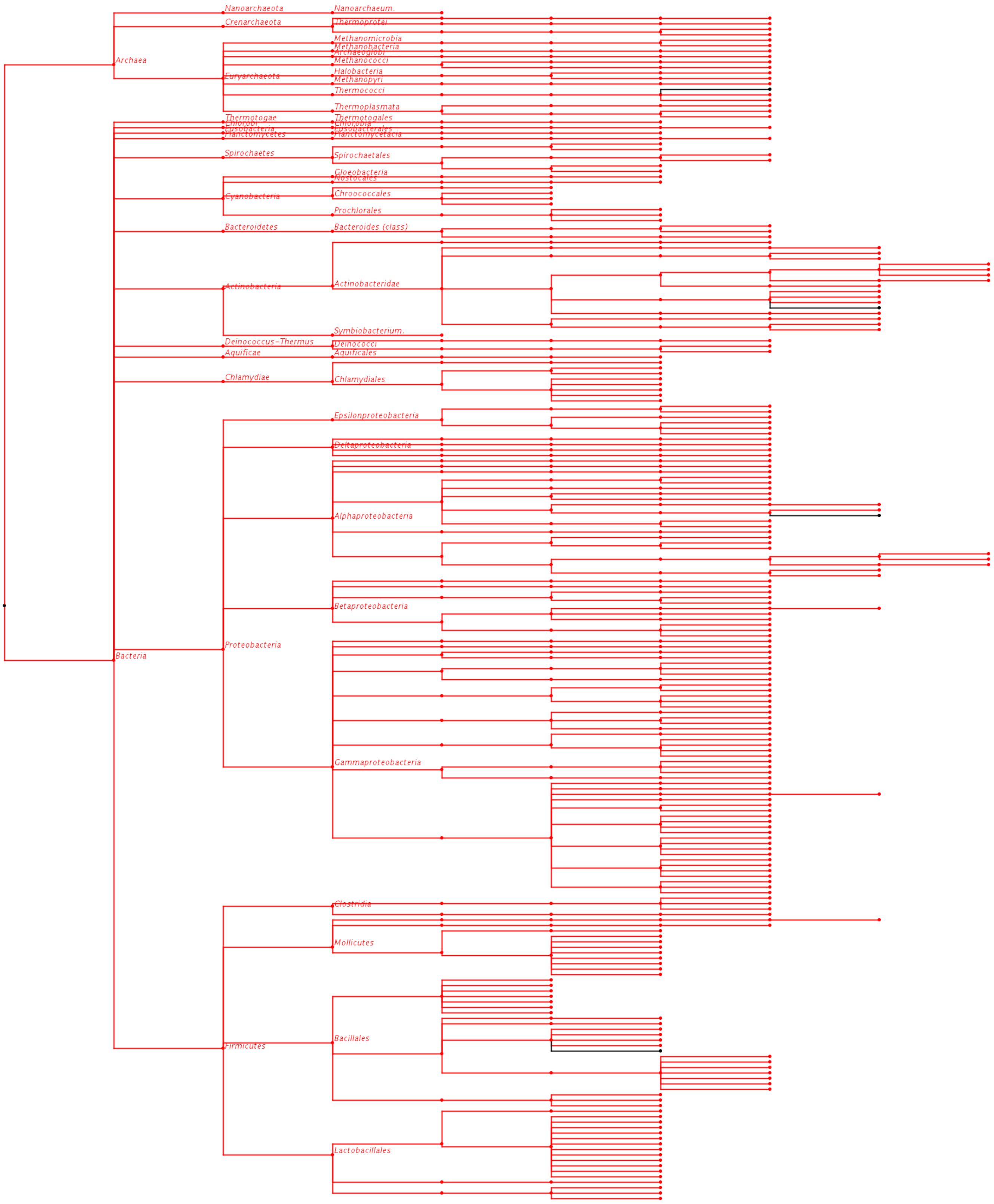

Level 9 = Nearest 7 = All

Acinetobacter sp.
Aeropyrum pernix
Agrobacterium tumefaciens
Anabaena sp.
Anaplasma marginale
Aquifex aeolicus
Archaeoglobus fulgidus
Azoarcus sp.
Bacillus anthracis
Bacillus anthracis
Bacillus anthracis
Bacillus cereus
Bacillus cereus
Bacillus cereus
Bacillus clausii
Bacillus halodurans
Bacillus licheniformis
Bacillus subtilis
Bacillus thuringiensis
Bacteroides fragilis
Bacteroides thetaiotaomicron
Bartonella henselae
Bartonella quintana
Bdellovibrio bacteriovorus
Bifidobacterium longum
Bordetella bronchiseptica
Bordetella parapertussis
Bordetella pertussis
Borrelia burgdorferi
Borrelia garinii
Bradyrhizobium japonicum
Brucella melitensis
Brucella suis
Buchnera aphidicola
Buchnera aphidicola
Buchnera aphidicola
Burkholderia mallei
Burkholderia pseudomallei
Campylobacter jejuni
Campylobacter jejuni
Candidatus Blochmannia
Caulobacter crescentus
Chlamydia muridarum
Chlamydia pneumoniae
Chlamydia pneumoniae
Chlamydia pneumoniae
Chlamydia pneumoniae
Chlamydia trachomatis
Chlamydophila caviae
Chlorobium tepidum
Chromobacterium violaceum
Clostridium acetobutylicum
Clostridium perfringens
Clostridium tetani
Corynebacterium diphtheriae
Corynebacterium efficiens
Corynebacterium glutamicum
Coxiella burnetii
Deinococcus radiodurans
Desulfotalea psychrophila
Desulfovibrio vulgaris
Ehrlichia ruminantium
Ehrlichia ruminantium
Enterococcus faecalis
Erwinia carotovora
Escherichia coli
Escherichia coli
Escherichia coli
Escherichia coli
Francisella tularensis
Fusobacterium nucleatum
Geobacillus kaustophilus
Geobacter sulfurreducens
Gloeobacter violaceus
Gluconobacter oxydans
Haemophilus ducreyi
Haemophilus influenzae
Haloarcula marismortui
Halobacterium salinarium
Helicobacter hepaticus
Helicobacter pylori
Helicobacter pylori
Idiomarina loihiensis
Lactobacillus acidophilus
Lactobacillus johnsonii
Lactobacillus plantarum
Lactococcus lactis
Legionella pneumophila
Legionella pneumophila
Legionella pneumophila
Leifsonia xyli
Leptospira interrogans
Leptospira interrogans
Listeria innocua
Listeria monocytogenes
Listeria monocytogenes
Mannheimia succiniciproducens
Mesoplasma florum
Methanobacterium thermoautotrophicum
Methanococcus jannaschii
Methanococcus maripaludis
Methanopyrus kandleri
Methanosarcina acetivorans
Methanosarcina mazei
Methylococcus capsulatus
Mycobacterium bovis
Mycobacterium leprae
Mycobacterium paratuberculosis
Mycobacterium tuberculosis
Mycobacterium tuberculosis
Mycoplasma gallisepticum
Mycoplasma genitalium
Mycoplasma hyopneumoniae
Mycoplasma mobile
Mycoplasma mycoides
Mycoplasma penetrans
Mycoplasma pneumoniae
Mycoplasma pulmonis
Nanoarchaeum equitans
Neisseria meningitidis
Neisseria meningitidis
Nitrosomonas europaea
Nocardia farcinica
Oceanobacillus iheyensis
Onion yellows
Parachlamydia sp.
Pasteurella multocida
Photobacterium profundum
Photorhabdus luminescens
Picrophilus torridus
Porphyromonas gingivalis
Prochlorococcus marinus
Prochlorococcus marinus
Prochlorococcus marinus
Propionibacterium acnes
Pseudomonas aeruginosa
Pseudomonas putida
Pseudomonas syringae
Pyrobaculum aerophilum
Pyrococcus furiosus
Pyrococcus horikoshii
Ralstonia solanacearum
Rhizobium loti
Rhizobium meliloti
Rhodopirellula baltica
Rhodopseudomonas palustris
Rickettsia conorii
Rickettsia prowazekii
Rickettsia typhi
Salmonella paratyphi-a
Salmonella typhi
Salmonella typhi
Salmonella typhimurium
Shewanella oneidensis
Shigella flexneri
Shigella flexneri
Silicibacter pomeroyi
Staphylococcus aureus
Staphylococcus aureus
Staphylococcus aureus
Staphylococcus aureus
Staphylococcus aureus
Staphylococcus aureus
Staphylococcus epidermidis
Streptococcus agalactiae
Streptococcus agalactiae
Streptococcus mutans
Streptococcus pneumoniae
Streptococcus pneumoniae
Streptococcus pyogenes
Streptococcus pyogenes
Streptococcus pyogenes
Streptococcus pyogenes
Streptococcus pyogenes
Streptococcus thermophilus
Streptococcus thermophilus
Streptomyces avermitilis
Streptomyces coelicolor
Sulfolobus solfataricus
Sulfolobus tokodaii
Symbiobacterium thermophilum
Synechococcus elongatus
Synechococcus sp.
Synechococcus sp.
Synechocystis sp.
Thermoanaerobacter tengcongensis
Thermoplasma acidophilum
Thermoplasma volcanium
Thermotoga maritima
Thermus thermophilus
Thermus thermophilus
Treponema denticola
Treponema pallidum
Tropheryma whipplei
Tropheryma whipplei
Ureaplasma parvum
Vibrio cholerae
Vibrio parahaemolyticus
Vibrio vulnificus
Vibrio vulnificus
Wigglesworthia glossinidia
Wolbachia pipientis
Wolbachia sp.
Wolinella succinogenes
Xanthomonas axonopodis
Xanthomonas campestris
Xanthomonas oryzae
Xylella fastidiosa
Xylella fastidiosa
Yersinia pestis
Yersinia pestis
Yersinia pestis
Yersinia pseudotuberculosis
Zymomonas mobilis

Level 9 = Nearest 7 = All

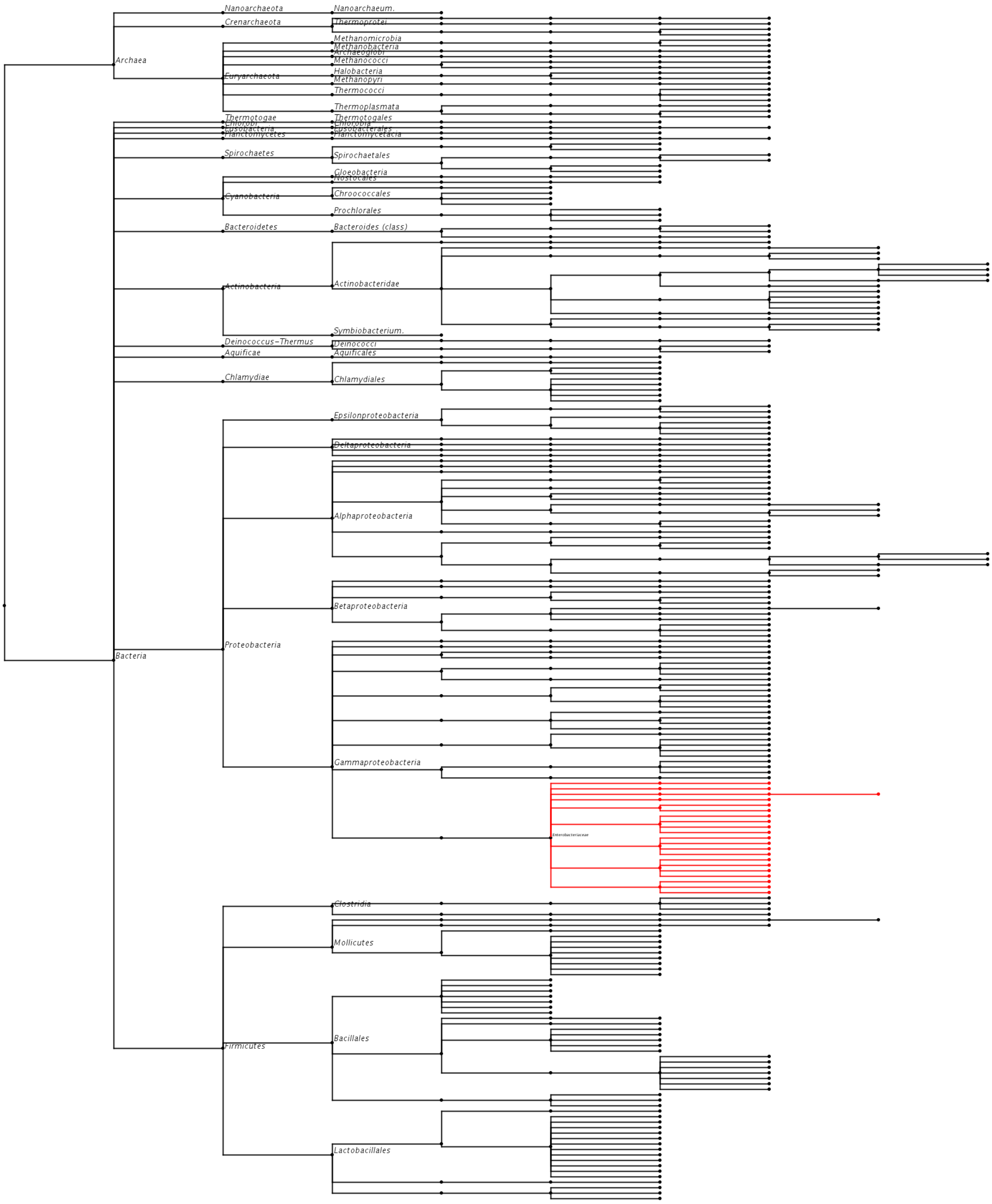
Nanoarchaeota
Nanoarchaeum.
Crenarchaeota
Thermoprotei
Archaea
Methanomicrobia
Methanobacteria
Archaeoglobi
Methanococci
Halobacteria
Euryarchaeota
Methanopyri
Thermococci
Thermoplasmata
Thermotogae
Thermotogales
Chlorobi
Chlorobia
Fusobacteria
Fusobacterales
Planctomycetes
Planctomycetacia
Spirochaetes
Spirochaetales
Gloeobacteria
Nostocales
Cyanobacteria
Chroococcales
Prochlorales
Bacteroidetes
Bacteroides (class)
Actinobacteria
Actinobacteridae
Symbiobacterium.
Deinococcus−Thermus
Deinococci
Aquificae
Aquificales
Chlamydiae
Chlamydiales
Epsilonproteobacteria
Deltaproteobacteria
Alphaproteobacteria
Betaproteobacteria
Bacteria
Proteobacteria
Gammaproteobacteria
Clostridia
Mollicutes
Bacillales
Firmicutes
Lactobacillales

Nearest 2

Buchnera aphidicola Acyrthosiphon pisum
Buchnera aphidicola Baizongia pistaciae
Buchnera aphidicola Schizaphis graminum
Candidatus Blochmannia floridanus
Erwinia carotovora
Escherichia coli EDL933
Escherichia coli K12
Escherichia coli O6 UPEC
Escherichia coli Sakai
Photorhabdus luminescens
Salmonella paratyphi-a
Salmonella typhi ATCC 700931
Salmonella typhi CT18
Salmonella typhimurium
Shigella flexneri 2457T
Shigella flexneri 301
Wigglesworthia glossinidia brevipalpis
Yersinia pestis 91001
Yersinia pestis CO-92
Yersinia pestis KIM5
Yersinia pseudotuberculosis

Nearest 2

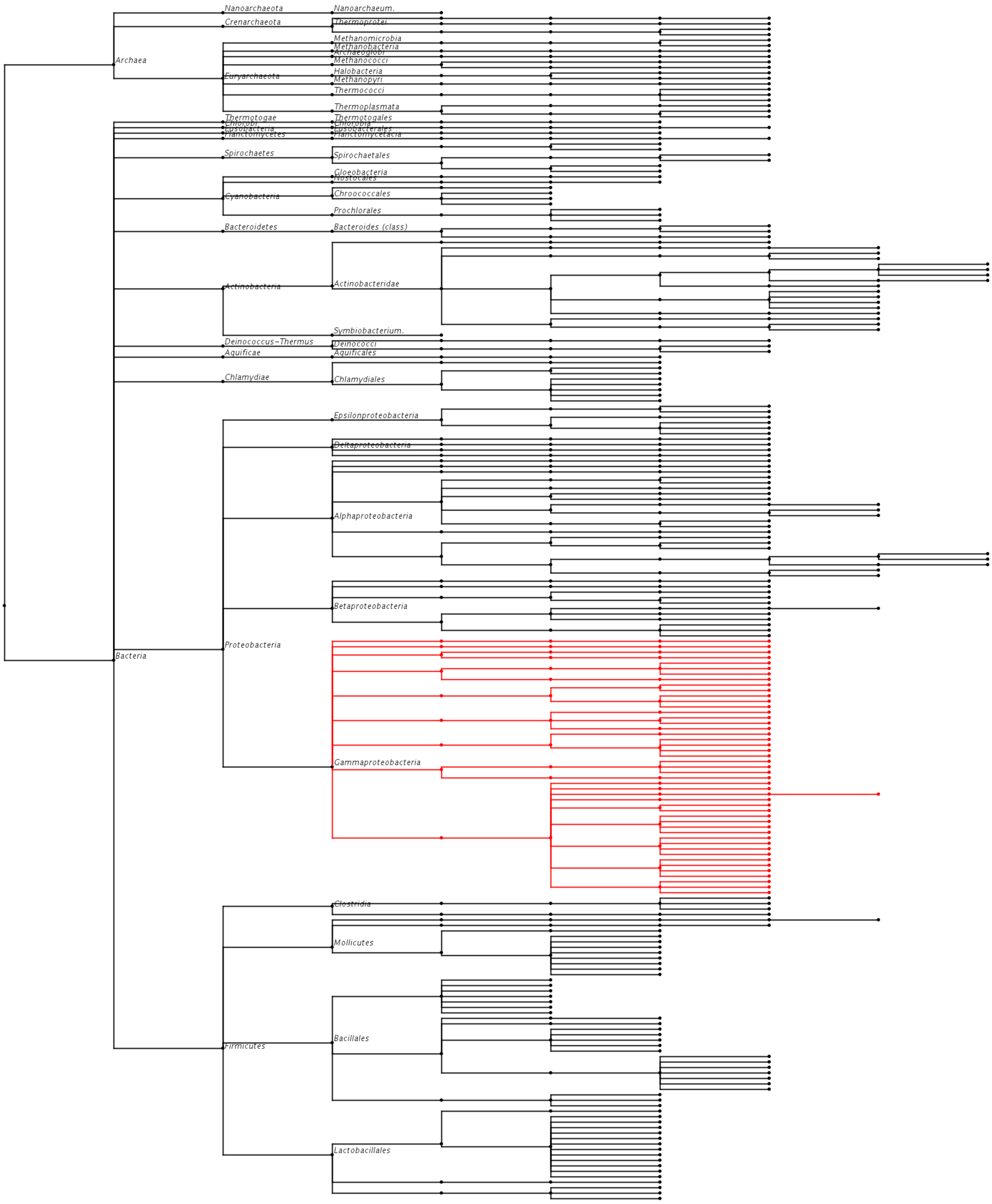

Nearest 3 = Nearest 4

Acinetobacter sp.
Buchnera aphidicola Acyrthosiphon pisum
Buchnera aphidicola Baizongia pistaciae
Buchnera aphidicola Schizaphis graminum
Candidatus Blochmannia floridanus
Coxiella burnetii
Erwinia carotovora
Escherichia coli EDL933
Escherichia coli K12
Escherichia coli O6 UPEC
Escherichia coli Sakai
Francisella tularensis
Haemophilus ducreyi
Haemophilus influenzae ATCC 51907
Idiomarina loihiensis
Legionella pneumophila Lens
Legionella pneumophila Paris
Legionella pneumophila Philadelphia 1
Mannheimia succiniciproducens
Methylococcus capsulatus
Pasteurella multocida
Photobacterium profundum
Photorhabdus luminescens
Pseudomonas aeruginosa
Pseudomonas putida
Pseudomonas syringae tomato
Salmonella paratyphi-a
Salmonella typhi ATCC 700931
Salmonella typhi CT18
Salmonella typhimurium
Shewanella oneidensis
Shigella flexneri 2457T
Shigella flexneri 301
Vibrio cholerae
Vibrio parahaemolyticus
Vibrio vulnificus CMCP6
Vibrio vulnificus YJ016
Wigglesworthia glossinidia brevipalpis
Xanthomonas axonopodis
Xanthomonas campestris campestris
Xanthomonas oryzae
Xylella fastidiosa 9a5c
Xylella fastidiosa Temecula1
Yersinia pestis 91001
Yersinia pestis CO-92
Yersinia pestis KIM5
Yersinia pseudotuberculosis

Nearest 3 = Nearest 4

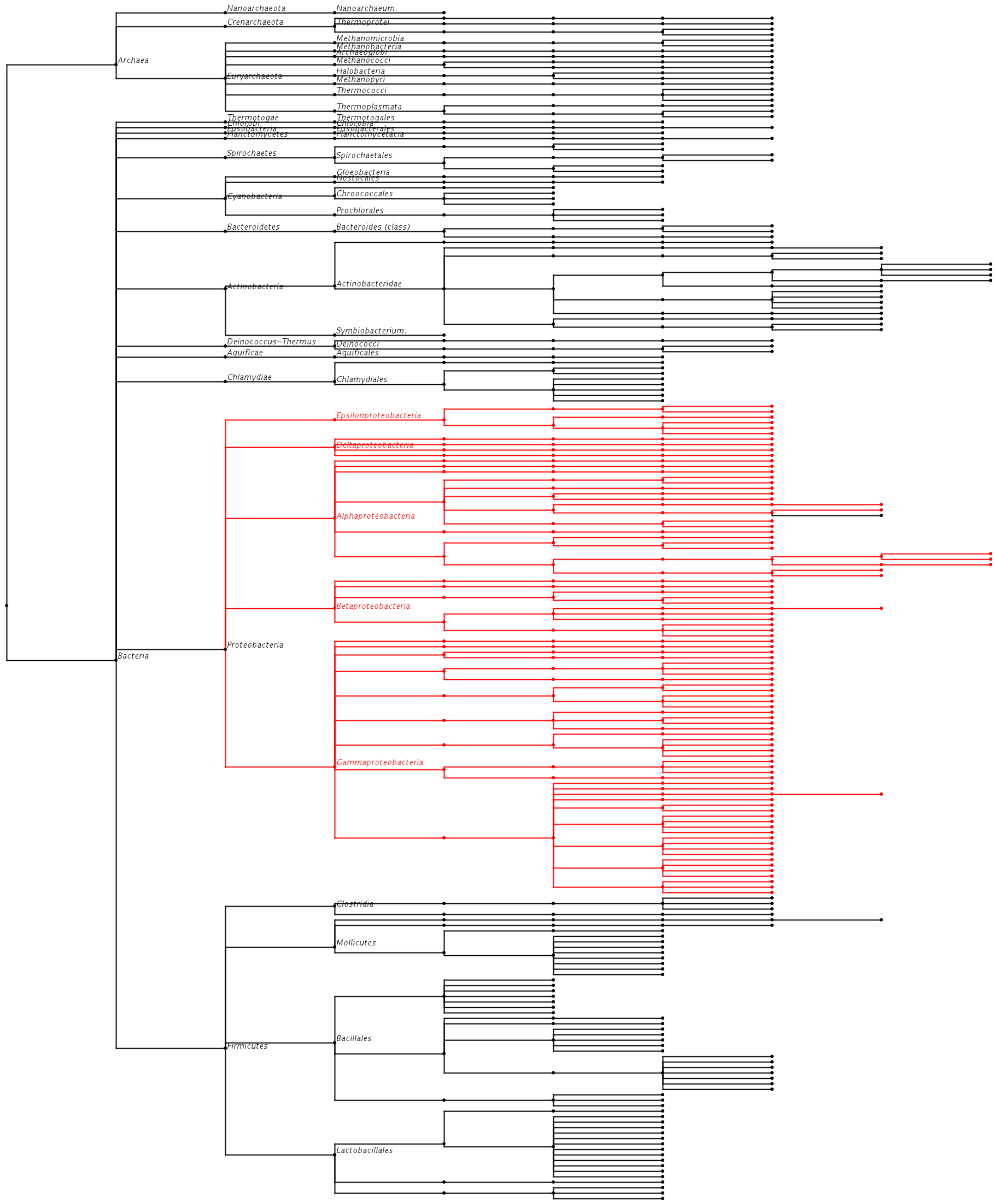
Archaea
Bacteria
Nanoarchaeota
Crenarchaeota
Euryarchaeota
Thermotogae
Chlorobi
Fusobacteria
Planctomycetes
Spirochaetes
Cyanobacteria
Bacteroidetes
Actinobacteria
Deinococcus−Thermus
Aquificae
Chlamydiae
Proteobacteria
Firmicutes
Nanoarchaeum.
Thermoprotei
Methanomicrobia
Methanobacteria
Archaeoglobi
Methanococci
Halobacteria
Methanopyri
Thermococci
Thermoplasmata
Thermotogales
Chlorobia
Fusobacteriales
Planctomycetacia
Spirochaetales
Gloeobacteria
Nostocales
Chroococcales
Prochlorales
Bacteroides (class)
Actinobacteridae
Symbiobacterium.
Deinococci
Aquificales
Chlamydiales
Epsilonproteobacteria
Deltaproteobacteria
Alphaproteobacteria
Betaproteobacteria
Gammaproteobacteria
Clostridia
Mollicutes
Bacillales
Lactobacillales

Nearest 5

Acinetobacter sp.
Agrobacterium tumefaciens Dupont
Anaplasma marginale
Azoarcus sp.
Bartonella henselae
Bartonella quintana
Bdellovibrio bacteriovorus
Bordetella bronchiseptica
Bordetella parapertussis
Bordetella pertussis
Bradyrhizobium japonicum
Brucella melitensis
Brucella suis
Buchnera aphidicola Acyrthosiphon pisum
Buchnera aphidicola Baizongia pistaciae
Buchnera aphidicola Schizaphis graminum
Burkholderia mallei
Burkholderia pseudomallei
Campylobacter jejuni NCTC 11168
Campylobacter jejuni RM1221
Candidatus Blochmannia floridanus
Caulobacter crescentus
Chromobacterium violaceum
Coxiella burnetii
Desulfotalea psychrophila
Desulfovibrio vulgaris
Ehrlichia ruminantium CIRAD
Ehrlichia ruminantium Gardel
Erwinia carotovora
Escherichia coli EDL933
Escherichia coli K12
Escherichia coli O6 UPEC
Escherichia coli Sakai
Francisella tularensis
Geobacter sulfurreducens
Gluconobacter oxydans
Haemophilus ducreyi
Haemophilus influenzae ATCC 51907
Helicobacter hepaticus
Helicobacter pylori ATCC 700392
Helicobacter pylori J99
Idiomarina loihiensis
Legionella pneumophila Lens
Legionella pneumophila Paris
Legionella pneumophila Philadelphia 1
Mannheimia succiniciproducens
Methylococcus capsulatus
Neisseria meningitidis A
Neisseria meningitidis B
Nitrosomonas europaea
Pasteurella multocida
Photobacterium profundum
Photorhabdus luminescens
Pseudomonas aeruginosa
Pseudomonas putida
Pseudomonas syringae tomato
Ralstonia solanacearum
Rhizobium loti
Rhizobium meliloti
Rhodopseudomonas palustris
Rickettsia conorii
Rickettsia prowazekii
Rickettsia typhi
Salmonella paratyphi-a
Salmonella typhi ATCC 700931
Salmonella typhi CT18
Salmonella typhimurium
Shewanella oneidensis
Shigella flexneri 2457T
Shigella flexneri 301
Silicibacter pomeroyi
Vibrio cholerae
Vibrio parahaemolyticus
Vibrio vulnificus CMCP6
Vibrio vulnificus YJ016
Wigglesworthia glossinidia brevipalpis
Wolbachia pipientis wMel
Wolbachia sp.
Wolinella succinogenes
Xanthomonas axonopodis
Xanthomonas campestris campestris
Xanthomonas oryzae
Xylella fastidiosa 9a5c
Xylella fastidiosa Temecula1
Yersinia pestis 91001
Yersinia pestis CO-92
Yersinia pestis KIM5
Yersinia pseudotuberculosis
Zymomonas mobilis

Nearest 5

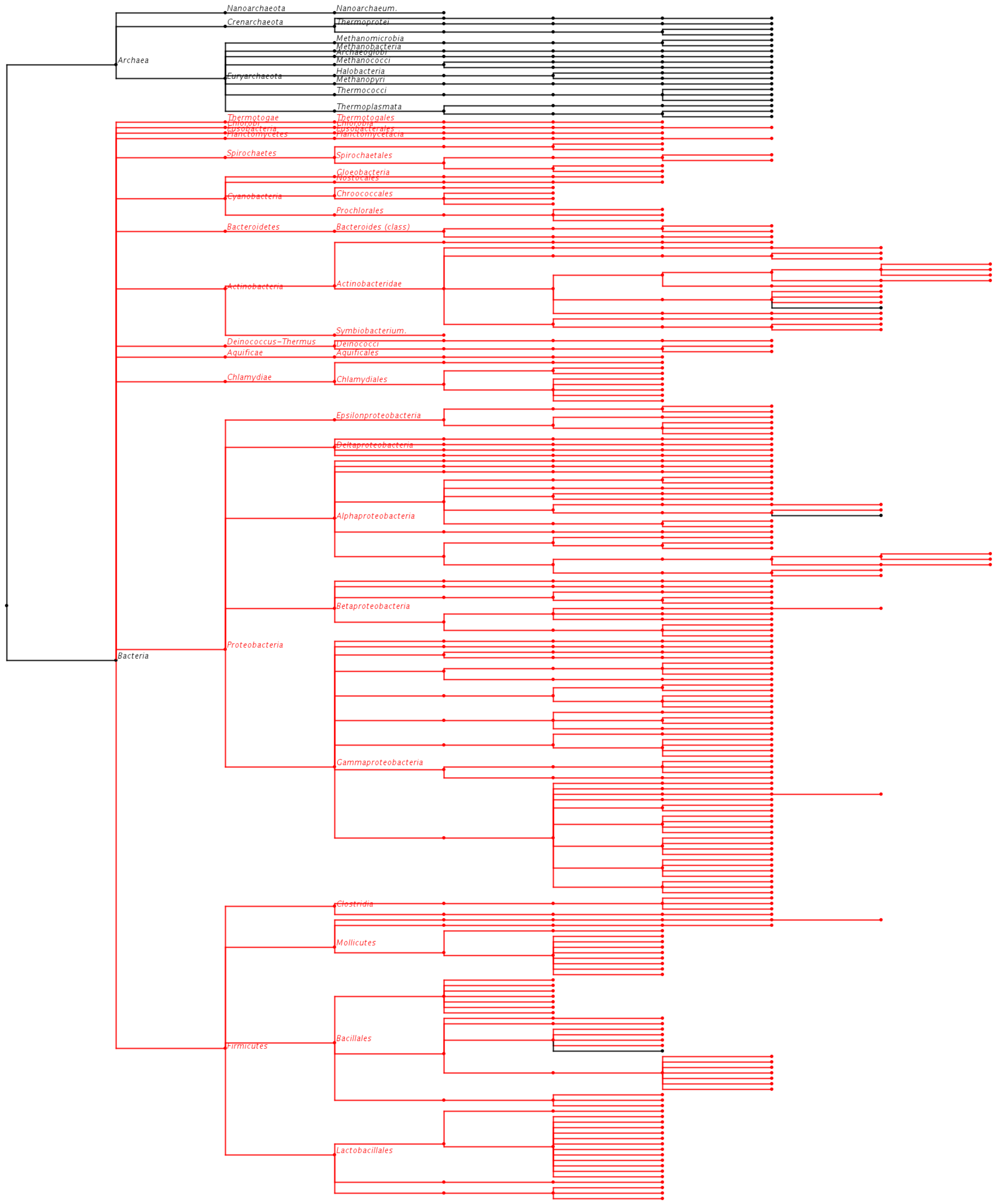

Archaea
Bacteria
Nanoarchaeota
Crenarchaeota
Euryarchaeota
Thermotogae
Chlorobi
Fusobacteria
Planctomycetes
Spirochaetes
Cyanobacteria
Bacteroidetes
Actinobacteria
Deinococcus−Thermus
Aquificae
Chlamydiae
Proteobacteria
Firmicutes
Nanoarchaeum.
Thermoprotei
Methanomicrobia
Methanobacteria
Archaeoglobi
Methanococci
Halobacteria
Methanopyri
Thermococci
Thermoplasmata
Thermotogales
Chlorobia
Fusobacteriales
Planctomycetacia
Spirochaetales
Gloeobacteria
Nostocales
Chroococcales
Prochlorales
Bacteroides (class)
Actinobacteridae
Symbiobacterium.
Deinococci
Aquificales
Chlamydiales
Epsilonproteobacteria
Deltaproteobacteria
Alphaproteobacteria
Betaproteobacteria
Gammaproteobacteria
Clostridia
Mollicutes
Bacillales
Lactobacillales

Nearest 6

Acinetobacter sp.
Agrobacterium tumefaciens Dupont
Anabaena sp.
Anaplasma marginale
Aquifex aeolicus
Azoarcus sp.
Bacillus anthracis 0581
Bacillus anthracis Porton
Bacillus anthracis Sterne
Bacillus cereus ATCC 10987
Bacillus cereus ATCC 14579
Bacillus cereus ZK
Bacillus clausii
Bacillus halodurans
Bacillus licheniformis Goettingen
Bacillus subtilis
Bacillus thuringiensis
Bacteroides fragilis YCH46
Bacteroides thetaiotaomicron
Bartonella henselae
Bartonella quintana
Bdellovibrio bacteriovorus
Bifidobacterium longum
Bordetella bronchiseptica
Bordetella parapertussis
Bordetella pertussis
Borrelia burgdorferi
Borrelia garinii
Bradyrhizobium japonicum
Brucella melitensis
Brucella suis
Buchnera aphidicola Acyrthosiphon pisum
Buchnera aphidicola Baizongia pistaciae
Buchnera aphidicola Schizaphis graminum
Burkholderia mallei
Burkholderia pseudomallei
Campylobacter jejuni NCTC 11168
Campylobacter jejuni RM1221
Candidatus Blochmannia floridanus
Caulobacter crescentus
Chlamydia muridarum
Chlamydia pneumoniae AR39
Chlamydia pneumoniae CWL029
Chlamydia pneumoniae J138
Chlamydia pneumoniae TW-183
Chlamydia trachomatis
Chlamydophila caviae
Chlorobium tepidum
Chromobacterium violaceum
Clostridium acetobutylicum
Clostridium perfringens
Clostridium tetani
Corynebacterium diphtheriae
Corynebacterium efficiens
Corynebacterium glutamicum Nakagawa
Coxiella burnetii
Deinococcus radiodurans
Desulfotalea psychrophila
Desulfovibrio vulgaris
Ehrlichia ruminantium CIRAD
Ehrlichia ruminantium Gardel
Enterococcus faecalis
Erwinia carotovora
Escherichia coli EDL933
Escherichia coli K12
Escherichia coli O6 UPEC
Escherichia coli Sakai
Francisella tularensis
Fusobacterium nucleatum
Geobacillus kaustophilus
Geobacter sulfurreducens
Gloeobacter violaceus
Gluconobacter oxydans
Haemophilus ducreyi
Haemophilus influenzae ATCC 51907
Helicobacter hepaticus
Helicobacter pylori ATCC 700392
Helicobacter pylori J99
Idiomarina loihiensis
Lactobacillus acidophilus
Lactobacillus johnsonii
Lactobacillus plantarum
Lactococcus lactis
Legionella pneumophila Lens
Legionella pneumophila Paris
Legionella pneumophila Philadelphia 1
Leifsonia xyli
Leptospira interrogans Icterohaemorrhagiae
Leptospira interrogans lai
Listeria innocua
Listeria monocytogenes 1/2a
Listeria monocytogenes 4b
Mannheimia succiniciproducens
Mesoplasma florum
Methylococcus capsulatus
Mycobacterium bovis
Mycobacterium leprae
Mycobacterium paratuberculosis
Mycobacterium tuberculosis H37Rv
Mycobacterium tuberculosis Oshkosh
Mycoplasma gallisepticum
Mycoplasma genitalium
Mycoplasma hyopneumoniae
Mycoplasma mobile
Mycoplasma mycoides
Mycoplasma penetrans
Mycoplasma pneumoniae
Mycoplasma pulmonis
Neisseria meningitidis A
Neisseria meningitidis B
Nitrosomonas europaea
Nocardia farcinica
Oceanobacillus iheyensis
Onion yellows phytoplasma
Parachlamydia sp.
Pasteurella multocida
Photobacterium profundum
Photorhabdus luminescens
Porphyromonas gingivalis
Prochlorococcus marinus CCMP 1375
Prochlorococcus marinus CCMP 1378
Prochlorococcus marinus MIT 9313
Propionibacterium acnes
Pseudomonas aeruginosa
Pseudomonas putida
Pseudomonas syringae tomato
Ralstonia solanacearum
Rhizobium loti
Rhizobium meliloti
Rhodopirellula baltica
Rhodopseudomonas palustris
Rickettsia conorii
Rickettsia prowazekii
Rickettsia typhi
Salmonella paratyphi-a
Salmonella typhi ATCC 700931
Salmonella typhi CT18
Salmonella typhimurium
Shewanella oneidensis
Shigella flexneri 2457T
Shigella flexneri 301
Silicibacter pomeroyi
Staphylococcus aureus COL
Staphylococcus aureus MRSA252
Staphylococcus aureus MSSA476
Staphylococcus aureus MW2
Staphylococcus aureus Mu50
Staphylococcus aureus N315
Staphylococcus epidermidis ATCC 12228
Streptococcus agalactiae III
Streptococcus agalactiae V
Streptococcus mutans
Streptococcus pneumoniae ATCC BAA-255
Streptococcus pneumoniae TIGR4
Streptococcus pyogenes MGAS10394
Streptococcus pyogenes MGAS315
Streptococcus pyogenes MGAS8232
Streptococcus pyogenes SF370
Streptococcus pyogenes SSI-1
Streptococcus thermophilus ATCC BAA-250
Streptococcus thermophilus CNRZ 1066
Streptomyces avermitilis
Streptomyces coelicolor
Symbiobacterium thermophilum
Synechococcus elongatus
Synechococcus sp. PCC 6301
Synechococcus sp. WH8102
Synechocystis sp.
Thermoanaerobacter tengcongensis
Thermotoga maritima
Thermus thermophilus HB27
Thermus thermophilus HB8
Treponema denticola
Treponema pallidum
Tropheryma whipplei TW08/27
Tropheryma whipplei Twist
Ureaplasma parvum
Vibrio cholerae
Vibrio parahaemolyticus
Vibrio vulnificus CMCP6
Vibrio vulnificus YJ016
Wigglesworthia glossinidia brevipalpis
Wolbachia pipientis wMel
Wolbachia sp.
Wolinella succinogenes
Xanthomonas axonopodis
Xanthomonas campestris campestris
Xanthomonas oryzae
Xylella fastidiosa 9a5c
Xylella fastidiosa Temecula1
Yersinia pestis 91001
Yersinia pestis CO-92
Yersinia pestis KIM5
Yersinia pseudotuberculosis
Zymomonas mobilis

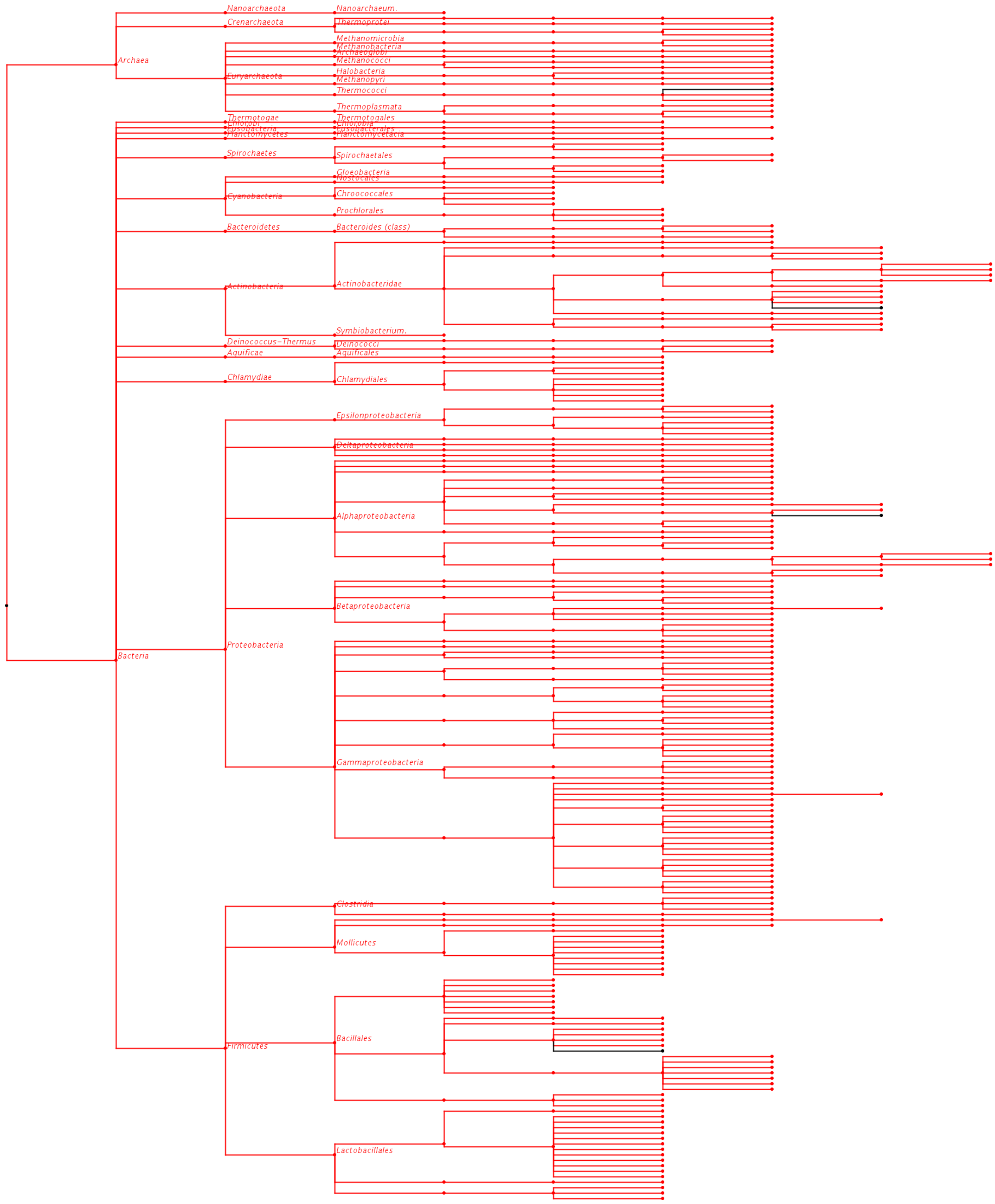

Nearest 7 = Level 9 = All

Acinetobacter sp.
Aeropyrum pernix
Agrobacterium tumefaciens
Anabaena sp.
Anaplasma marginale
Aquifex aeolicus
Archaeoglobus fulgidus
Azoarcus sp.
Bacillus anthracis
Bacillus anthracis
Bacillus anthracis
Bacillus cereus
Bacillus cereus
Bacillus cereus
Bacillus clausii
Bacillus halodurans
Bacillus licheniformis
Bacillus subtilis
Bacillus thuringiensis
Bacteroides fragilis
Bacteroides thetaiotaomicron
Bartonella henselae
Bartonella quintana
Bdellovibrio bacteriovorus
Bifidobacterium longum
Bordetella bronchiseptica
Bordetella parapertussis
Bordetella pertussis
Borrelia burgdorferi
Borrelia garinii
Bradyrhizobium japonicum
Brucella melitensis
Brucella suis
Buchnera aphidicola
Buchnera aphidicola
Buchnera aphidicola
Burkholderia mallei
Burkholderia pseudomallei
Campylobacter jejuni
Campylobacter jejuni
Candidatus Blochmannia
Caulobacter crescentus
Chlamydia muridarum
Chlamydia pneumoniae
Chlamydia pneumoniae
Chlamydia pneumoniae
Chlamydia pneumoniae
Chlamydia trachomatis
Chlamydophila caviae
Chlorobium tepidum
Chromobacterium violaceum
Clostridium acetobutylicum
Clostridium perfringens
Clostridium tetani
Corynebacterium diphtheriae
Corynebacterium efficiens
Corynebacterium glutamicum
Coxiella burnetii
Deinococcus radiodurans
Desulfotalea psychrophila
Desulfovibrio vulgaris
Ehrlichia ruminantium
Ehrlichia ruminantium
Enterococcus faecalis
Erwinia carotovora
Escherichia coli
Escherichia coli
Escherichia coli
Escherichia coli
Francisella tularensis
Fusobacterium nucleatum
Geobacillus kaustophilus
Geobacter sulfurreducens
Gloeobacter violaceus
Gluconobacter oxydans
Haemophilus ducreyi
Haemophilus influenzae
Haloarcula marismortui
Halobacterium salinarium
Helicobacter hepaticus
Helicobacter pylori
Helicobacter pylori
Idiomarina loihiensis
Lactobacillus acidophilus
Lactobacillus johnsonii
Lactobacillus plantarum
Lactococcus lactis
Legionella pneumophila
Legionella pneumophila
Legionella pneumophila
Leifsonia xyli
Leptospira interrogans
Leptospira interrogans
Listeria innocua
Listeria monocytogenes
Listeria monocytogenes
Mannheimia succiniciproducens
Mesoplasma florum
Methanobacterium thermoautotrophicum
Methanococcus jannaschii
Methanococcus maripaludis
Methanopyrus kandleri
Methanosarcina acetivorans
Methanosarcina mazei
Methylococcus capsulatus
Mycobacterium bovis
Mycobacterium leprae
Mycobacterium paratuberculosis
Mycobacterium tuberculosis
Mycobacterium tuberculosis
Mycoplasma gallisepticum
Mycoplasma genitalium
Mycoplasma hyopneumoniae
Mycoplasma mobile
Mycoplasma mycoides
Mycoplasma penetrans
Mycoplasma pneumoniae
Mycoplasma pulmonis
Nanoarchaeum equitans
Neisseria meningitidis
Neisseria meningitidis
Nitrosomonas europaea
Nocardia farcinica
Oceanobacillus iheyensis
Onion yellows
Parachlamydia sp.
Pasteurella multocida
Photobacterium profundum
Photorhabdus luminescens
Picrophilus torridus
Porphyromonas gingivalis
Prochlorococcus marinus
Prochlorococcus marinus
Prochlorococcus marinus
Propionibacterium acnes
Pseudomonas aeruginosa
Pseudomonas putida
Pseudomonas syringae
Pyrobaculum aerophilum
Pyrococcus furiosus
Pyrococcus horikoshii
Ralstonia solanacearum
Rhizobium loti
Rhizobium meliloti
Rhodopirellula baltica
Rhodopseudomonas palustris
Rickettsia conorii
Rickettsia prowazekii
Rickettsia typhi
Salmonella paratyphi-a
Salmonella typhi
Salmonella typhi
Salmonella typhimurium
Shewanella oneidensis
Shigella flexneri
Shigella flexneri
Silicibacter pomeroyi
Staphylococcus aureus
Staphylococcus aureus
Staphylococcus aureus
Staphylococcus aureus
Staphylococcus aureus
Staphylococcus aureus
Staphylococcus epidermidis
Streptococcus agalactiae
Streptococcus agalactiae
Streptococcus mutans
Streptococcus pneumoniae
Streptococcus pneumoniae
Streptococcus pyogenes
Streptococcus pyogenes
Streptococcus pyogenes
Streptococcus pyogenes
Streptococcus pyogenes
Streptococcus thermophilus
Streptococcus thermophilus
Streptomyces avermitilis
Streptomyces coelicolor
Sulfolobus solfataricus
Sulfolobus tokodaii
Symbiobacterium thermophilum
Synechococcus elongatus
Synechococcus sp.
Synechococcus sp.
Synechocystis sp.
Thermoanaerobacter tengcongensis
Thermoplasma acidophilum
Thermoplasma volcanium
Thermotoga maritima
Thermus thermophilus
Thermus thermophilus
Treponema denticola
Treponema pallidum
Tropheryma whipplei
Tropheryma whipplei
Ureaplasma parvum
Vibrio cholerae
Vibrio parahaemolyticus
Vibrio vulnificus
Vibrio vulnificus
Wigglesworthia glossinidia
Wolbachia pipientis
Wolbachia sp.
Wolinella succinogenes
Xanthomonas axonopodis
Xanthomonas campestris
Xanthomonas oryzae
Xylella fastidiosa
Xylella fastidiosa
Yersinia pestis
Yersinia pestis
Yersinia pestis
Yersinia pseudotuberculosis
Zymomonas mobilis

Nearest 7 = Level 9 = All
