# Supplementary material for: Selection of organisms for the co-evolution-based study of protein interactions
Source: BMC Bioinformatics. 2011 Sep 12;12:363. doi: 10.1186/1471-2105-12-363 (PMC3179974; doi:10.1186/1471-2105-12-363)
Supplement: Additional file 2 — Version of the Figure 2 with all plots in the same scale. [file 1471-2105-12-363-S2.PDF]

Additional file 2 - Version of the Figure 2 with all ROC plots in the same scale. The legend is the same as in Figure 2.

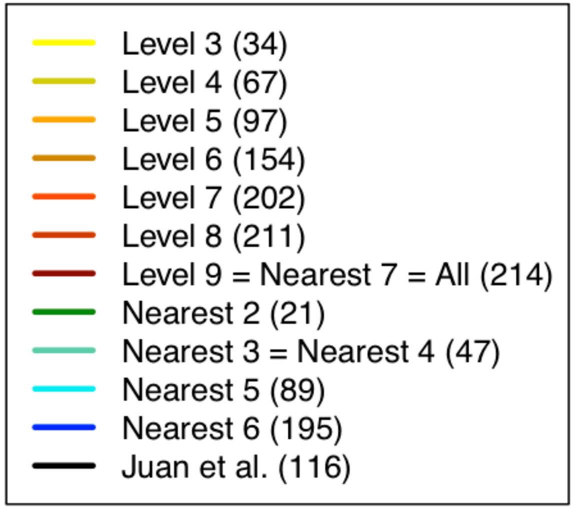

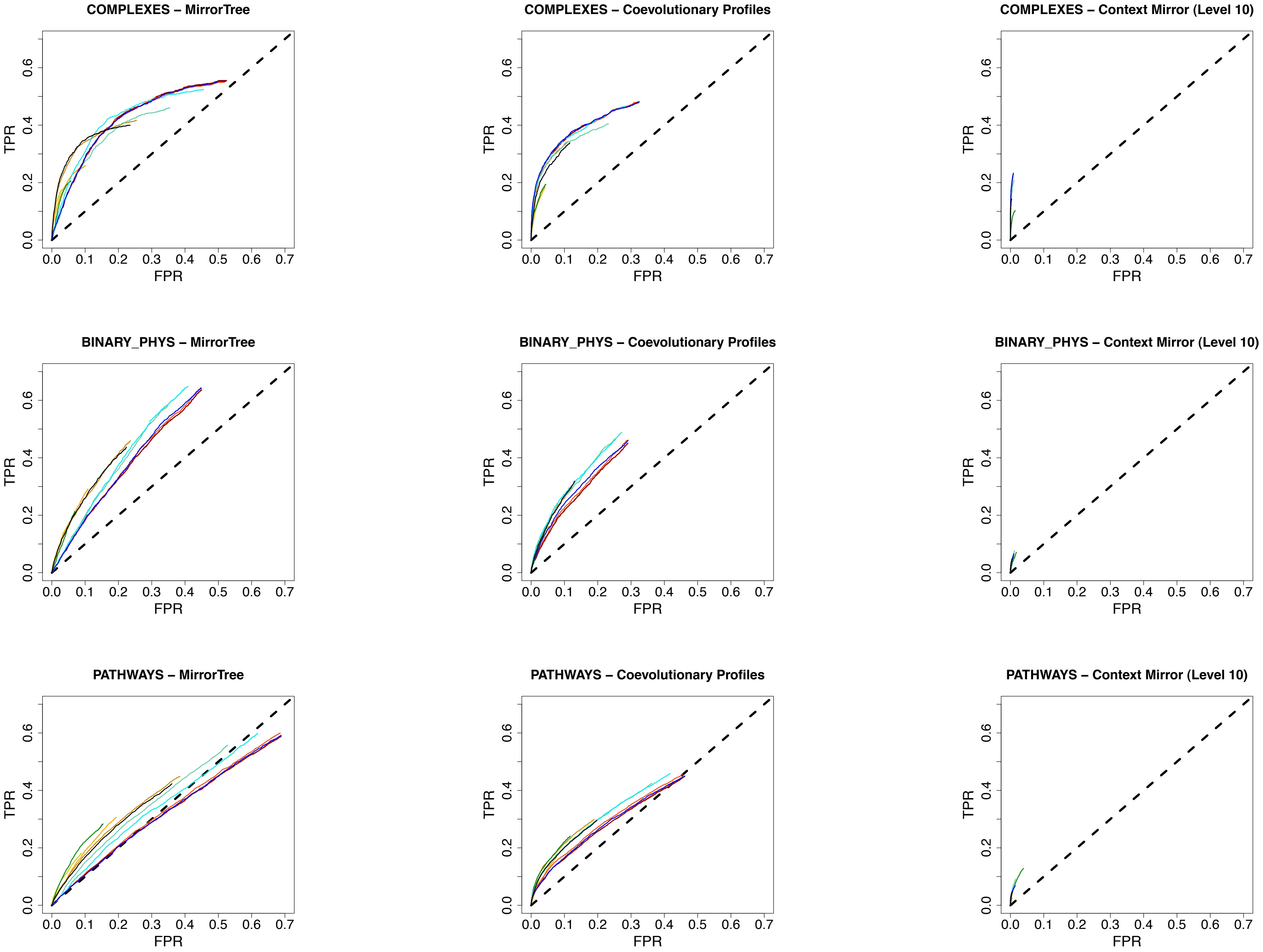

COMPLEXES – MirrorTree
COMPLEXES – Coevolutionary Profiles
COMPLEXES – Context Mirror (Level 10)
BINARY_PHYS – MirrorTree
BINARY_PHYS – Coevolutionary Profiles
BINARY_PHYS – Context Mirror (Level 10)
PATHWAYS – MirrorTree
PATHWAYS – Coevolutionary Profiles
PATHWAYS – Context Mirror (Level 10)
TPR
FPR
0.0
0.2
0.4
0.6
0.0 0.1 0.2 0.3 0.4 0.5 0.6 0.7
