# Supplementary material for: Selection of organisms for the co-evolution-based study of protein interactions
Source: BMC Bioinformatics. 2011 Sep 12;12:363. doi: 10.1186/1471-2105-12-363 (PMC3179974; doi:10.1186/1471-2105-12-363)
Supplement: Additional file 3 — Results of Figure 2 given in terms of F-measure (the harmonic mean between "precision" and "recall"). [file 1471-2105-12-363-S3.PDF]

Additional file 3 – Results in terms of F-measure *vs.* score of the method.

Another possible way of evaluating the capacity of a method in separating positives and negatives (as well as recovering the positives) is using “precision” and “recall”. Cutting the sorted list of scores at a given threshold, “precision” (also known as “positive predictive value” - PPV) and “recall” (the same as “sensitivity” and “true positive rate” (TPR) in binary classification) are defined as

$$\textit{precision} = Tp/(Tp+Fp)$$
$$\textit{recall} = Tp/P$$

where *Tp* and *Fp* are the true positives and false positives obtained at a that threshold, and *P* the total number of positives (irrespective of whether the method could be applied to them or not).

It is common to combine “precision” and recall into a single parameter called “F-measure”, which is the harmonic average of these two figures,

$$\textit{F-measure} = 2\cdot \textit{precision}\cdot \textit{recall}/(\textit{precision}+\textit{recall})$$

And to represent the F-measure against the threshold (score of the method, correlation coefficient in this case).

These F-measure *vs.* score plots complement the ROC plots of Figure 2 in providing information on the different performance of the methods and organisms datasets.

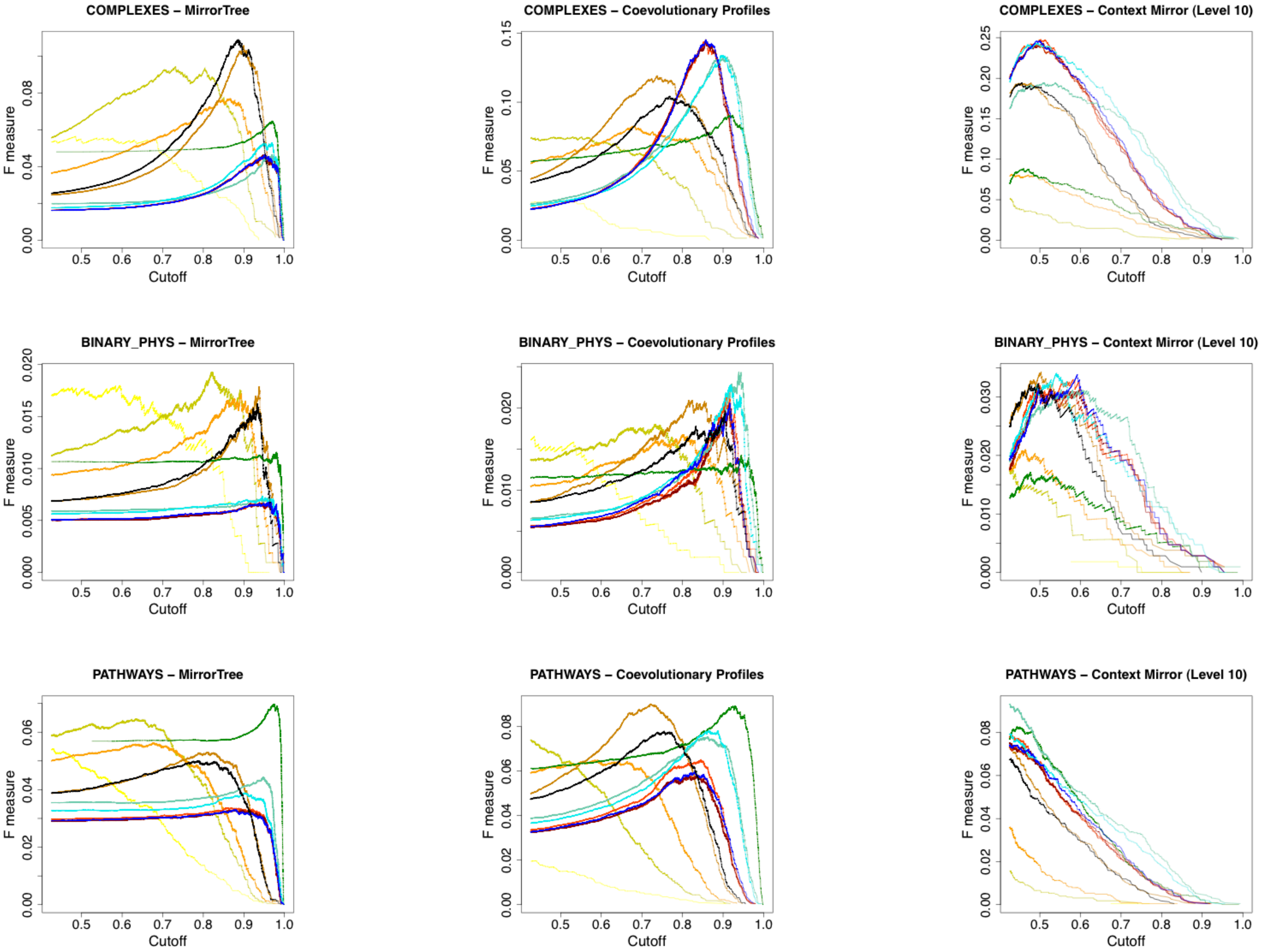
COMPLEXES – MirrorTree
COMPLEXES – Coevolutionary Profiles
COMPLEXES – Context Mirror (Level 10)
BINARY_PHYS – MirrorTree
BINARY_PHYS – Coevolutionary Profiles
BINARY_PHYS – Context Mirror (Level 10)
PATHWAYS – MirrorTree
PATHWAYS – Coevolutionary Profiles
PATHWAYS – Context Mirror (Level 10)
F measure
Cutoff
0.5 0.6 0.7 0.8 0.9 1.0
0.00 0.04 0.08
0.00 0.05 0.10 0.15
0.00 0.05 0.10 0.15 0.20 0.25
0.000 0.005 0.010 0.015 0.020
0.000 0.010 0.020
0.000 0.010 0.020 0.030
0.00 0.02 0.04 0.06
0.00 0.02 0.04 0.06 0.08
