# Supplementary material for: Selection of organisms for the co-evolution-based study of protein interactions
Source: BMC Bioinformatics. 2011 Sep 12;12:363. doi: 10.1186/1471-2105-12-363 (PMC3179974; doi:10.1186/1471-2105-12-363)
Supplement: Additional file 4 — Maximum of the F-measure curves of "Additional file 3". This parameter can be regarded as a single numerical estimator of the performance of a given method/organism set, although it does not encompass all the information of a ROC curve. [file 1471-2105-12-363-S4.PDF]

Additional file 4 – Maximum F-measure for each method/organism set.

As a single numerical estimator or performance for a given method using a given dataset of organisms, we show here the maximum of the F-measure (See “Additional file 3”). The legend is the same as in Figure 2, Additional File 2 and Additional file 3.

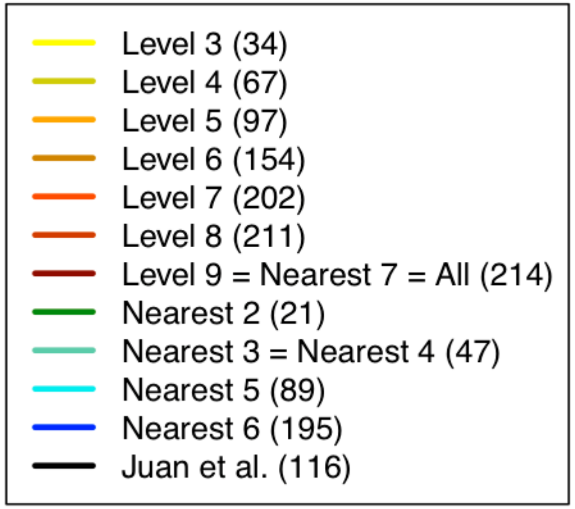

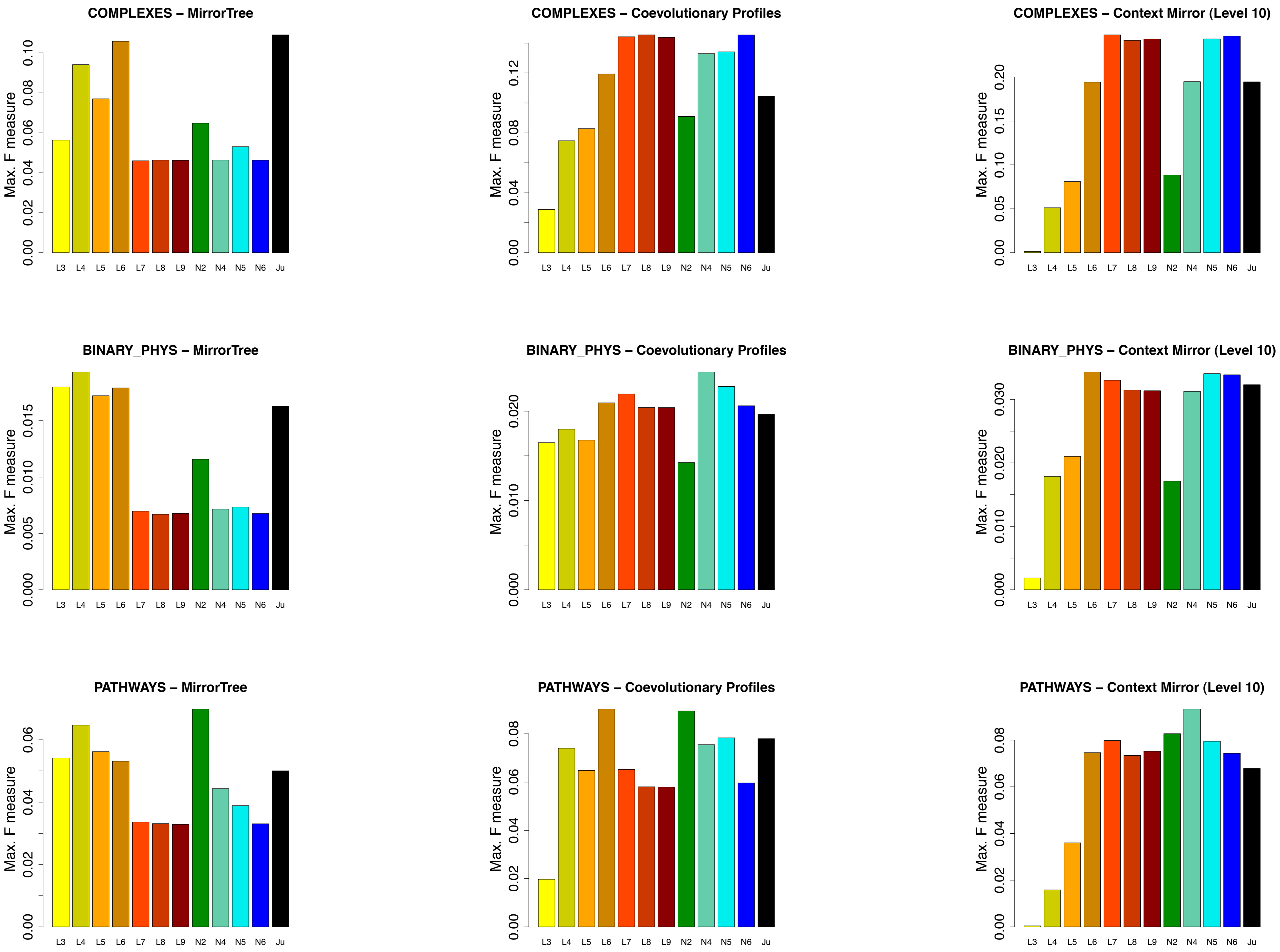
COMPLEXES – MirrorTree
COMPLEXES – Coevolutionary Profiles
COMPLEXES – Context Mirror (Level 10)
BINARY_PHYS – MirrorTree
BINARY_PHYS – Coevolutionary Profiles
BINARY_PHYS – Context Mirror (Level 10)
PATHWAYS – MirrorTree
PATHWAYS – Coevolutionary Profiles
PATHWAYS – Context Mirror (Level 10)
Max. F measure
L3 L4 L5 L6 L7 L8 L9 N2 N4 N5 N6 Ju
